# Neurodevelopment in Children Exposed to Zika *in utero*: Clinical and Molecular Aspects

**DOI:** 10.3389/fgene.2022.758715

**Published:** 2022-03-08

**Authors:** Lavínia Schuler-Faccini, Miguel del Campo, Alfredo García-Alix, Liana O. Ventura, Juliano André Boquett, Vanessa van der Linden, André Pessoa, Hélio van der Linden Júnior, Camila V. Ventura, Mariana Carvalho Leal, Thayne Woycinck Kowalski, Lais Rodrigues Gerzson, Carla Skilhan de Almeida, Lucélia Santi, Walter O. Beys-da-Silva, André Quincozes-Santos, Jorge A. Guimarães, Patricia P. Garcez, Julia do Amaral Gomes, Fernanda Sales Luiz Vianna, André Anjos da Silva, Lucas Rosa Fraga, Maria Teresa Vieira Sanseverino, Alysson R. Muotri, Rafael Lopes da Rosa, Alberto Mantovani Abeche, Clairton Marcolongo-Pereira, Diogo O. Souza

**Affiliations:** ^1^ Universidade Federal do Rio Grande do Sul, UFRGS, Porto Alegre, Brazil; ^2^ Medical Genetics Service, Hospital de Clinicas de Porto Alegre, HCPA, Porto Alegre, Brazil; ^3^ Department of Pediatrics, School of Medicine, University of California San Diego, and Rady Children’s Hospital San Diego, San Diego, CA, United States; ^4^ Neonatal Neurology, NeNe Foundation, Madrid, Spain; ^5^ Department of Ophthalmology, Fundação Altino Ventura, FAV, Recife, Brazil; ^6^ Hospital Barão de Lucena, Recife, Brazil; ^7^ Hospital Infantil Albert Sabin, Fortaleza, Brazil; ^8^ Universidade Estadual do Ceará, Fortaleza, Brazil; ^9^ Dr. Henrique Santillo Rehabilitation and Readaptation Center, Goiânia, Brazil; ^10^ Universidade Federal de Pernambuco, UFPE, Recife, Brazil; ^11^ CESUCA—Centro Universitário, Cachoeirinha, Brazil; ^12^ Universidade Federal do Rio de Janeiro, Rio de Janeiro, Brazil; ^13^ School of Medicine, Graduate Program in Medical Sciences–Universidade do Vale do Taquari–UNIVATES, Lajeado, Brazil; ^14^ School of Medicine, Universidade do Vale do Rio dos Sinos–UNISINOS, São Leopoldo, Brazil; ^15^ Pontifícia Universidade Católica do Rio Grande do Sul, PUCRS, Porto Alegre, Brazil; ^16^ Centro Universitário do Estírito Santo, UNESC, Colatina, Brazil

**Keywords:** microcephaly, zika (ZIKV), epilepsy, cerebral palsy, neurodevelopement, eye

## Abstract

Five years after the identification of Zika virus as a human teratogen, we reviewed the early clinical manifestations, collectively called congenital Zika syndrome (CZS). Children with CZS have a very poor prognosis with extremely low performance in motor, cognitive, and language development domains, and practically all feature severe forms of cerebral palsy. However, these manifestations are the tip of the iceberg, with some children presenting milder forms of deficits. Additionally, neurodevelopment can be in the normal range in the majority of the non-microcephalic children born without brain or eye abnormalities. Vertical transmission and the resulting disruption in development of the brain are much less frequent when maternal infection occurs in the second half of the pregnancy. Experimental studies have alerted to the possibility of other behavioral outcomes both in prenatally infected children and in postnatal and adult infections. Cofactors play a vital role in the development of CZS and involve genetic, environmental, nutritional, and social determinants leading to the asymmetric distribution of cases. Some of these social variables also limit access to multidisciplinary professional treatment.

## Introduction

Although the Zika Virus (ZIKV) was first identified in monkeys in Uganda in 1947, and later in humans, it has received little attention since the clinical manifestations of its infection was comparatively mild relatively to other flavivirus, notably yellow fever and dengue viruses (Macnamara, 1954). In late 2015, however, the consequences of prenatal infection by the Zika virus were suspected based on the occurrence of a cluster of children born with microcephaly in Northeast Brazil (França et al., 2016; Schuler-Faccini et al., 2016). The associaton between congenital zika infection and microcephaly was soon confirmed (Rasmussen et al., 2016) and the increase in congenital microcephaly cases followed outbreaks of zika infection outbreaks in other Latin American countries (Morris et al., 2021).

The five major components of the phenotype used to define CZS were external physical dysmorphic features, a pattern of neurologic anomalies, joint contractures, neuroimaging findings, and ocular and hearing abnormalities (Moore et al., 2017; del Campo et al., 2017). Here, we review the phenotypes associated with prenatal ZIKV infection and the neurodevelopment in these affected children. We also cover the experimental data about the cellular and molecular mechanisms underlying these clinical outcomes.

### Literature Search

Literature searches were performed in the Ovid-Medline, PubMed, EMBASE, SciELO, and Cochrane databases. The search terms “Zika”, “ZIKV”, “congenital Zika syndrome”, “CZS” and “Zika neuro*” were used. There were no language restrictions. The final reference list was generated based on the relevance to the topics covered in this review. Studies published until 29 March 2021 were included. To ensure the full coverage of the literature, additional screenings of the reference lists of important reviews and eligible studies were performed to discover any research articles that were not identified in the initial database searches.

## Congenital Zika Syndrome

In those studies in which descriptions of the physical and neuroimaging phenotypes are thorough, including case control studies, two groups of children were identified. Those exposed to ZIKV *in utero* that did not have any abnormal phenotypic physical or neurologic findings, and those that had severe and complex physical and neurological phenotypes with devastating consequences for later development, a spectrum which was called the Congenital Zika syndrome (Moore et al., 2017; del Campo et al., 2017). In some studies, in which isolated microcephaly was reported, an incomplete description of other phenotypic features may underlie this false appearance of a mild isolated phenotype.

### Microcephaly and Dysmorphic Features

**FIGURE 1:**
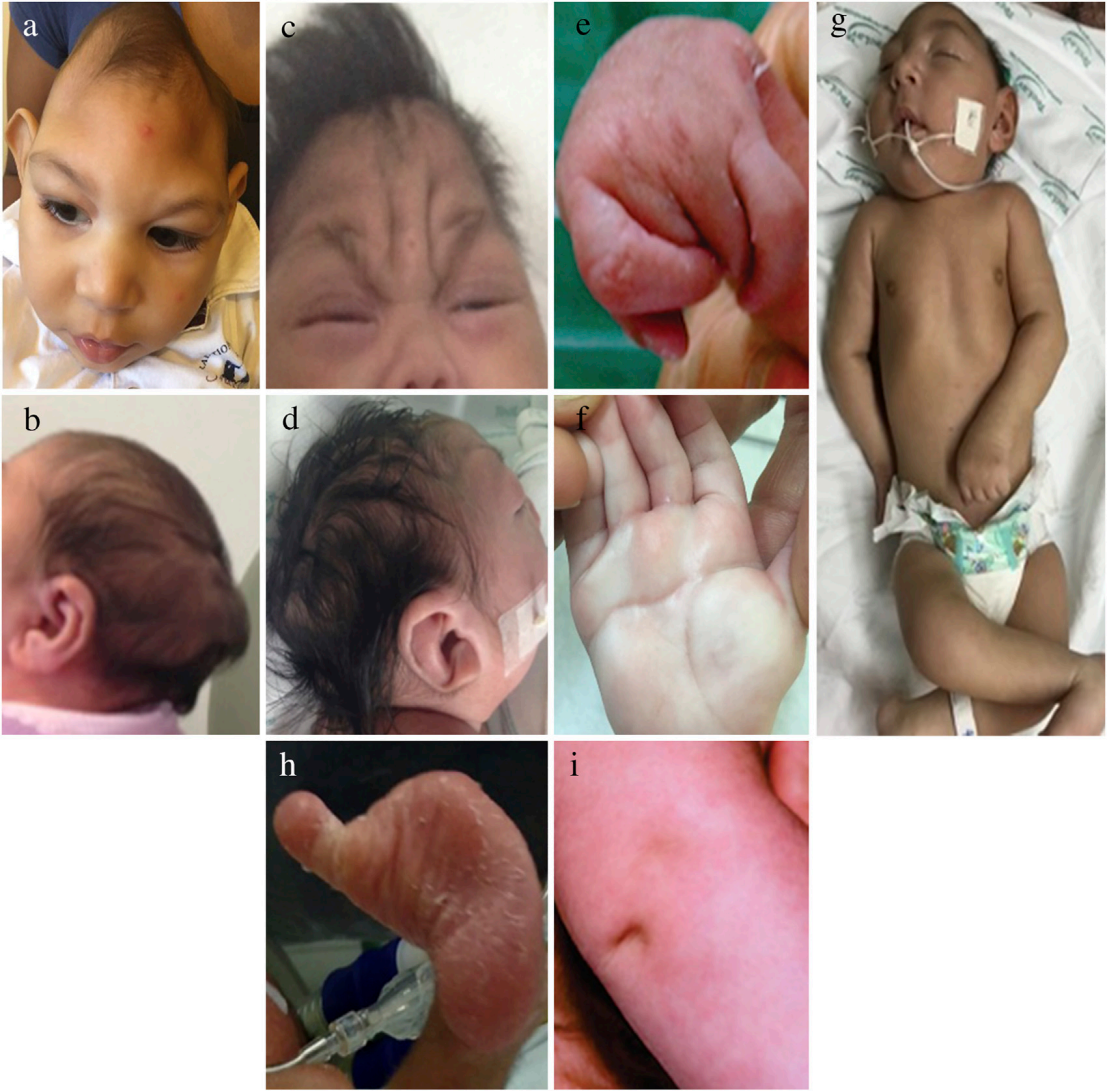
Dysmorphic features in the CZS. Dysmorphic physical features characteristic of the CZS. One year old boy with craniofacial disproportion, and marked reduction of the volume of the cranial vault, including lateral depressions of the frontal bone **(A)** and an occipital prominence **(B)**. These severe features have remained visible since birth. Skin redundance in frontal and glabellar area with vertical folds in a 3 month old **(C)** and multiple folds across the scalp, called *cutis rugata* in a 1 month old **(D)**. Generalized contractures of large and small joints **(E)**. Contractures of all fingers **(F)**. Abnormal single transverse palmar crease **(G)**. Club foot **(H)**. Deep dimple at the knee **(I)**.

Figure 1 illustrates the main craniofacial findings in babies affected by CZS, including a small head circumference (HC) with markedly decreased volume of the cranial vault in disproportion with a normal face (Figure 1A), abnormally shaped skulls (Figures 1A,B), and redundant scalp (Figures 1C,D) which were the main dysmorphic findingsreported in the majority of cases since the initial case series in the literature (Moore et al., 2017; del Campo et al., 2017; van der Linden et al., 2016a; Aragao et al., 2016). Craniofacial disproportion (Figure 1A). Microcephaly was described in 87.3% of 48 cases, 7 and in 75.4% in 87 cases in Brazil (del Campo et al., 2017; Moura da Silva et al., 2016) and the anomaly was severe (<−3Z scores below the mean) in more than 50% of cases in both studies. Postnatal microcephaly in the first months of life was also reported (Moura da Silva et al., 2016; van der Linden et al., 2018). Case control studies have later shown somewhat similar numbers, 69% born with microcephaly among infants and fetuses with birth defects in the US (Honein et al., 2017). In the US territories, among all cases that had microcephaly, 20/84 (24%) were born with normal head circumference but developed it after birth (Rice et al., 2018).

A “collapsed” skull appearance and redundant scalp had been previously described as the foetal brain disruption sequence (FBDS), a dysmorphology term used to define the disruption of the development of the brain resulting in the visible phenotypic morphological changes in the skull and scalp (Russel et al., 1984). An abrupt decrease in intracranial hydrostatic pressure was suggested to underlie the apparent collapse of the skull (Russel et al., 1984). Generalized arthrogryposis is a serious complication exhibited by up to 7% of children with CZS as shown in Figure 1E. Predominantly distal arthrogryposis is present in greater than 10% of children, involving only or mainly the smaller joints of the wrists, ankles, hands and feet as shown in Figures 1A,F,H and previously described by del Campo et al. (2017), Moura da Silva et al. (2016), and van der Linden et al. (2016b) amongst others. The club foot and other abnormal foot positions, restriction of hand movements, and flexion contractures of the fingers (camptodactilies) are frequently seen associated with CZS (del Campo et al., 2017).

### Early Neurological Phenotypes

**FIGURE 2:**
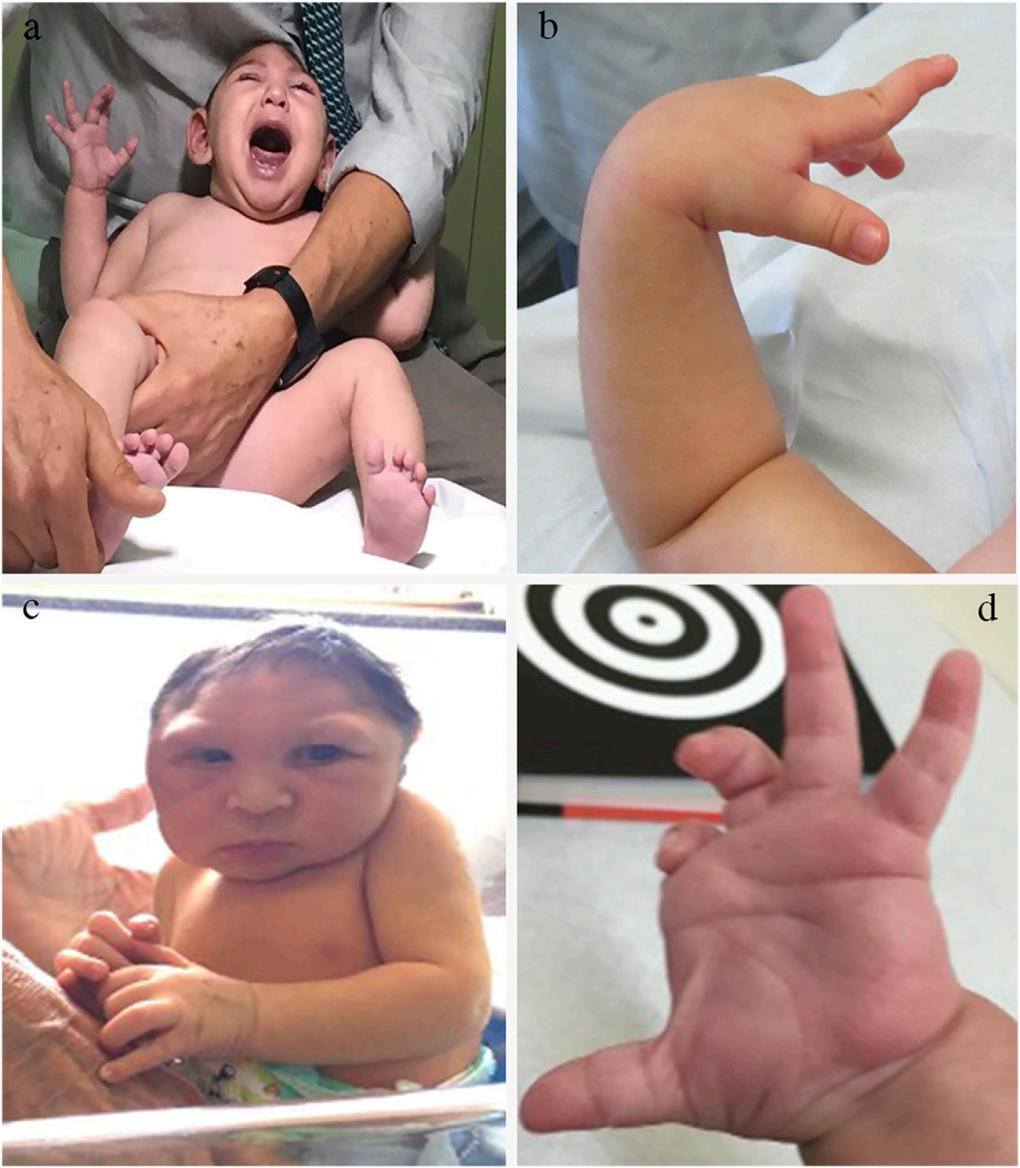
Neurological examination findings in the CZS. Common findings in the neurological examination of infants with CZS. **(A)** Irritability with constant inconsolable cry. **(B)** Swan neck position of the second finger caused by dystonic hyperextension. **(C)** Marked hypertonia leading to an almost stable sitting position right after birth. **(D)** Dystonic position of the fingers, with flexion of 2–3 and extension of 3–4.

Besides irritability, sometimes unconsolable (Figure 2A), the primary and more consistent signs associated with CZS during the first months of life were hypertonia, spasticity, and hyperreflexia (Figure 2C), reflecting the involvement of the primary motor system (motor cortex and corticospinal tract) in almost all affected newborns and infants (Moore et al., 2017; del Campo et al., 2017) This is coupled with etrapiramidal signs such as dystonia and dyskinetic movements (Figures 2B,D) The peripheral motor neurons may be affected, leading to predominant hypotonia and weakness in some cases (van der Linden et al., 2016b; Aragao et al., 2017). Brainstem cranial nerves are often affected, leading to oromotor dysfunction, persistent swallowing disorders and respiratory problems (Pereira et al., 2020). Diaphragmatic paralysis due to the phrenic nerve dysfunction was reported in 3 of 120 (3.6%) infants with CZS with the need for assisted ventilation, and led to early death (van der Linden et al., 2019a).

Although the pyramidal signs are predominant during the first year, dystonic movements and postures are prominent during the second year. In a single-centre cohort of 32 patients with CZS, dystonic movements and postures were observed in 95.2% of the cases (van der Linden et al., 2020a). The high association of movement disorders, neuromuscular findings and pyramidal signs in those patients resulted in the diagnosis of the combined form of cerebral palsy with predominant corticospinal or neuromuscular manifestations (Pereira et al., 2020). In addition, children between 34 and 40 months of age had persisting primitive reflexes in 90.5% of the cases (van der Linden et al., 2020a). Deficits in the higher neurological function are reflected by excessive crying with poor consolability, minimal contact with the environment and abnormally weak responses to stimuli (del Campo et al., 2017).

### Structural Brain Anomalies

**FIGURE 3:**
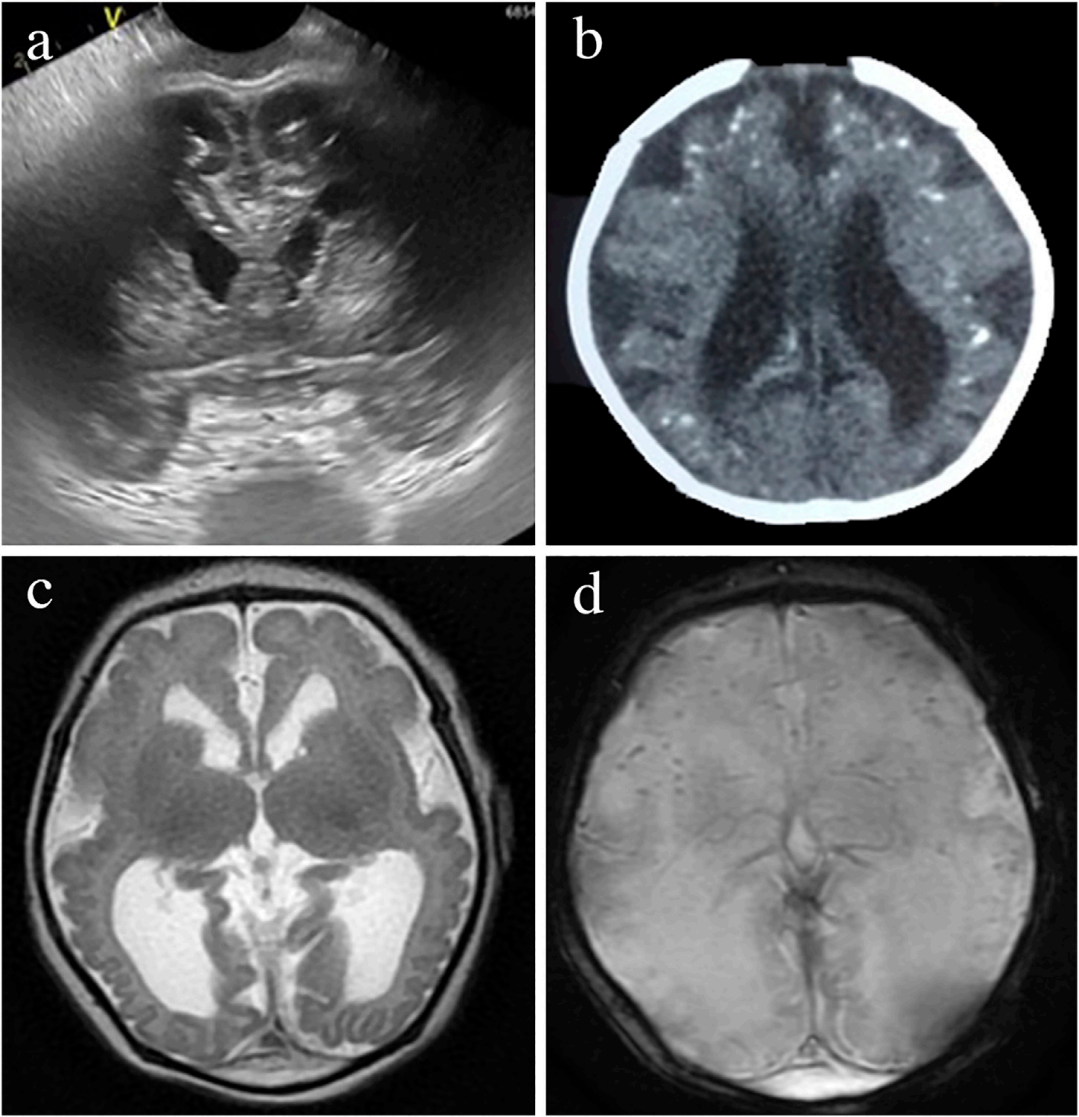
Brain neuroimaging studies in an infant with microcephaly and prenatal Zika infection. Head ultrasound image of a prefrontal coronal plane showing dilated frontal horns and cerebral calcifications below the subcortical border **(A)**. CT axial image showing ventriculomegaly, calcifications predominantly located in the cortical-subcortical border and simplified gyral pattern **(B)**. MRI- T2-weigthed axial image showing a very simplified frontal gyral pattern, ventriculomegaly, germinolytic pseudocysts in the caudothalamic groove, an open operculum, reduced white matter volume and absence of myelin in the posterior limb of the internal capsule **(C)**. Gradient Echo T2* axial MRI image showing cerebral calcifications seeb as small, rounded, homogeneous, dark dot-like lesions **(D)**.

Brain abnormalities are the key manifestations of CZS. Most cases are easily identified on cranial ultrasound (CUS) shown in Figure 3A, computed tomography (CT) in Figure 3B, or magnetic resonance imaging (MRI) in Figures 3C,D, although each technique will better identify some of the findings. The least invasive and most available neuroimaging approach in every clinical setting should be favored at present, including foetal or neonatal ultrasound as the first option (Adebanjo et al., 2017).

**FIGURE 4:**
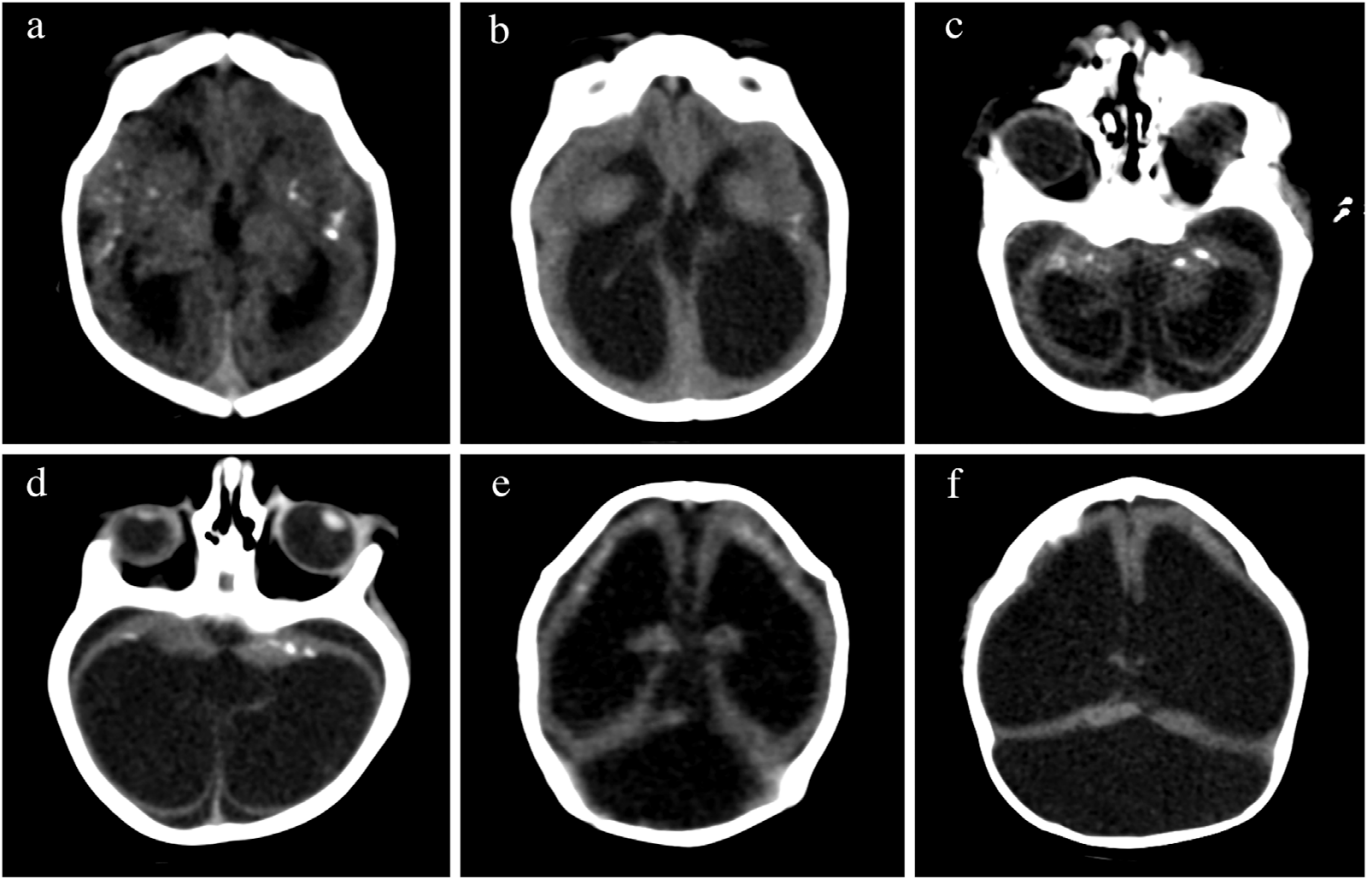
Changes in brain imaging observed during the first year of life. CT of the brain of the same patient in the first month of life in the upper row in **(A,C,E)**, at 6 months of age in lower rows **(B,D)**, and at 13 months of age in **(F)**. Calcifications are more difficult to visualize at 6 months of age **(B,D)** than at birth **(A,B)**. We observed increases in volume of intraventricular and extra-axial fluids (bottom pictures) in comparison to the initial exam (top pictures). The increasing ventricular dilatation did not show clinical or imaging signs of increased intracranial pressure at 6 months **(B,D)**, but a high tentorium confirmed progressive hydrocephalus requiring surgical shunting at 13 months **(F)**.

Although none of the imaging findings alone is exclusive of CZS, the combination of calcifications at the junction of the cortical and subcortical white matter and in the basal ganglia, with a thin cortex, simplified gyral patterns, ventriculomegaly, and increased extra-axial fluid is observed in more than 90% of the infants with CZS (Aragao et al., 2106; Soares de Oliveira-Szejnfeld et al., 2016; Cavalheiro et al., 2016; Sanz Cortes et al., 2018) as shown in Figures 3, 4. The number of calcifications varies, their distribution is scant and the pattern is sparse to multiple and coalescent (Pereira et al., 2020; Soares de Oliveira-Szejnfeld et al., 2016) Malformations of the cortical development, including polymicrogyria, white matter anomalies, thin corpus callosum, and cerebellar and brain stem hypoplasias, may be present and will be better identified by MRI as shown in Figures 3C,D and reported elsewhere (Soares de Oliveira-Szejnfeld et al., 2016; Sanz Cortes et al., 2018).

There are very few studies examining the changes of the neuroimaging abnormalities over time in infants with CZS. Some children developed progressive enlargement of the ventricles (Figures 4B,D) and severe progressive hydrocephalus that required the placement of a ventriculoperitoneal shunt (Figure 4F) also seen in other studies (van der Linden et al., 2019b). One longitudinal study showed that the calcifications might be reduced or even not detected on the images at 1 year of age, hence recommending the need for neuroimaging soon after birth when CZS is suspected (Aragao et al., 2017). These calcifications were more difficult to identify at 6 months of age (Figures 4C,D) than at birth (Figures 4A,B) in some of the cases evaluated by the authors of this manuscript.

The majority of exposed children without abnormal clinical dysmorphological and neurological symptoms at birth had normal brain imaging and were developmentally normal, as we discuss later (Cardoso et al., 2019; Einspieler et al., 2019; Valdes et al., 2019; Gerzson et al., 2020; Sobral da Silva et al., 2020; Grant et al., 2021). In a population study in Martinique, all babies from the mothers with the ZIKV infection who were clinically normal at birth were examined by MRI. None of the infants showed brain injuries attributable to the ZIKV, and all had normal myelination, gyration, and brain parenchyma for their age (Mejdoubi et al., 2017). This observation is consistent with other studies of neonates with intrauterine ZIKV exposure who were normocephalic at birth (Mulkey et al., 2018; Sobral da Silva et al., 2020). A single study found that one-third of infants that appeared clinically normal at birth presented nonspecific postnatal neuroimaging findings, such as lenticulostriate vasculopathy, germinolityc or subependimal cysts, and had lower scores on the motor examination (Mulkey et al., 2019). Therefore, additional data are needed to confirm whether the infants with normal MRI early may have abnormal imaging findings later. If they develop abnormal clinical outcomes the information on the recurrence needs to be elucidated as well.

### Electroclinical Profile

Epilepsy is a frequent finding in CZS. According to a recent meta-analysis with 903 patients, the overall rate of epilepsy in children with CZS was estimated at 60% [95% confidence interval (CI) 0.51—0.68] (van der Linden et al., 2018). The epileptic spasms were the primary type of seizures during the first year of life (72%), while focal seizures were more common in the second year (van der Linden et al., 2018), and the frequency of epilepsy varies with age, experiencing higher rates in older children. Epilepsy usually presents in the initial months of life (mean age 4.9 months) (van der Linden et al., 2018). Electroencephalogram (EEG) demonstrates severe abnormal activities in 94% of the cases, with slow and disorganized tracings in most patients (van der Linden et al., 2018). Focal (51%) and multifocal (44%) epileptiform discharges were the critical findings of the EEG. Although identified less often, hypsarrhythmia (11%) and burst suppression (8%) patterns were associated with a higher risk of drug resistance (van der Linden et al., 2018). Abnormal background (100%) and focal epileptiform activity (54%) were the most frequent findings after the first year of life in children with CZS (Maia et al., 2021). In a series of 55 children, a pattern of continuous or almost continuous epileptiform discharges during sleep was seen in 40% of the cases, and it was correlated with the presence of subcortical calcifications and multifocal epileptiform discharges from the previous EEG showing (van der Linden et al., 2020b). The response to anti-epileptic drugs is generally low, ranging from only 20% seizure control in the first year to 30% seizure control in the second year (Maia et al., 2021).

A recent study cross sectional study including 43 children with CZS examining the relationship between epilepsy and structural neuroimaging findings found that the EEG characteristics were correlated with the degree of neuroimaging abnormalities (Nunes et al., 2021). The group of patients with mild neuroimaging findings showed a greater probability of having more frequently normal EEG sleep patterns and no interictal epileptogenic activity. Another recent study has reported that certain findings on cranial CT associate a greater probability of developing epilepsy (Sequerra et al., 2020). Smaller brain volumes were associated with epilepsy and the presence of interictal epileptiform discharges as well as with impaired development of sleep spindles, in the first year of life. The presence of rhombencephalon malformation was associated with higher risk of epilepsy.

### Visual and Hearing Findings

**FIGURE 5:**
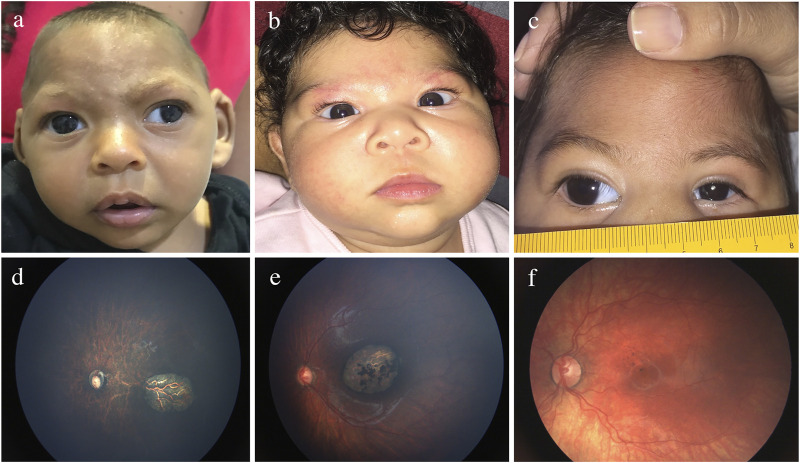
Eye findings in children with prenatal Zika infection. **(A)** Exotropia. **(B)** Esotropia. **(C)** Microphthalmia. **(D)** Optic nerve hypoplasia. **(E)** Chorio-retinal scarring. **(F)** Pigmentary macular mottling.

Although the mechanisms of ocular pathogenesis in CZS are not completely elucidated, experimental studies suggest that the viral spread to the eye can be haematogenous, through the inner and outer blood-retinal barriers, and/or the axonal along the visual tract (Fernandez et al., 2017). Figure 5 and Supplementary Table S1 shows the wide range of ocular manifestations in children with CZS (Ventura et al., 2018; de Paula Freitas et al., 2016; Ventura et al., 2016a; Zin et al., 2017; de Paula Freitas et al., 2017a; de Paula Freitas et al., 2017b; Ventura et al., 2016b; Ventura et al., 2021).

Structural ocular findings can be present in up to 55% of infants, and although not totally specific, they are distinguishable from the manifestations of other types of prenatal infection (Ventura et al., 2016a; de Paula Freitas et al., 2016; Yepez et al., 2017; Zin et al., 2017; Ventura et al., 2018). The frequent characteristics are well-defined chorioretinal scars and pigmentary mottling located in the macula (Ventura et al., 2016a; Ventura et al., 2016b; de Paula Freitas et al., 2016; de Paula Freitas et al., 2017a; de Paula Freitas et al., 2017b; Yepez et al., 2017; Zin et al., 2017; Ventura et al., 2018). Optic nerve abnormalities are present in approximately one-third of the patients and consist of optic disk hypoplasia, and pallor and increased cupping (Ventura et al., 2016a; Ventura et al., 2016b; de Paula Freitas et al., 2016; de Paula Freitas et al., 2017a; de Paula Freitas et al., 2017b; Yepez et al., 2017; Zin et al., 2017; Ventura et al., 2018). Abnormal retinal vasculature, including vasculature attenuation, avascularity, increased tortuosity, and haemorrhages, may be present (Ventura et al., 2019). Since these vascular anomalies may be associated with tractional retinal detachment, lifelong clinical surveillance is crucial. Anterior segment structural abnormalities such as microphthalmia, microcornea, congenital glaucoma, congenital cataract, iris coloboma, and lens subluxation have been reported (de Paula Freitas et al., 2016; de Paula Freitas et al., 2017a; de Paula Freitas et al., 2017b). Hypoaccomodation and significant refractive errors are also seen, requiring correction with spectacles for better visual development (Ventura et al., 2017; Ventura et al., 2018). Ocular manifestations with severe microcephaly are more frequently observed in newborns infected with the ZIKV during the first trimester of pregnancy (Ventura et al., 2016a), but one study detected ocular abnormalities in 42% of children without microcephaly (Zin et al., 2017). Therefore, comprehensive ocular evaluation is recommended for all children born to ZIKV-exposed mothers.

The impairment of the visual performance in children with CZS is a result of ocular abnormalities but also due to the associated CNS damage (Ventura et al., 2018). Initial studies reported visual impairment in 90–100% and severe or worse visual impairment in 72% of children with CZS (Ventura et al., 2016b; Ventura et al., 2018). In one study, 85% of the children affected with CZS and normal ocular examinations had visual impairment, and all children with CZS had abnormal visual evoked potentials (Ventura et al., 2018). These studies support the diagnosis of cortical visual impairment in children with CZS. As a consequence, early strabismus and nystagmus are far more frequent than in neurologically typical children (Ventura et al., 2016b; Ventura et al., 2018). Therefore, visual rehabilitation is of fundamental importance to maximally enhance the residual vision (Ventura et al., 2018; Ventura et al., 2019).

Although few studies evaluated hearing in the ZIKV-exposed infants beyond neonatal screenings, sensorineural hearing loss (SNHL) appears to be infrequent and occurs in infants with microcephaly and other severe anomalies of CZS (Leal et al., 2016). Among 70 children with laboratory-confirmed ZIKV infection and microcephaly in Brazil, 5.8% presented hearing loss (SNHL) (Faria et al., 2020). This percentage was 3.8% among 78 prospectively ascertained infants likely exposed to the ZIKV *in utero* (Faria et al., 2020). Although we predicted that the conductive hearing loss secondary to otitis, upper airway congestion, and infections would be increased in these infants, no data are yet available.

### Differential Diagnosis

The combination of brain and ocular defects leading to severe disabilities and sensory deficits seen in CZS is also encountered in other neurotropic congenital infections by *Toxoplasma gondii*, *Other infections, Cytomegalovirus*, *Rubella*, and *Herpes simplex* virus known by the acronym TORCH, (Khan and Khan, 2018). The phenotypes caused by ZIKV infection, including microcephaly, are found more consistently and are more severe in CZS than in other TORCH infections. While most of the key neuroimaging findings of CZS are far more severe and complex than those caused by other TORCH infections, severe neurological abnormalities may be seen in cases of congenital CMV (Alarcon et al., 2013). The combination of calcifications at the junction of the cortical and subcortical white matter with ventriculomegaly and abnormal simplified gyral pattern are highly suggestive of CZS, whereas a periventricular distribution of the calcifications is suggestive of CMV (Aragao et al., 2016). In contrast to other TORCH infections, no active ocular inflammation has been reported in CZS. Optic nerve hypoplasia and pigmentary mottling outside of the areas of chorioretinal atrophy together are typical of CZS and are usually not seen in the other TORCH. Additionally, CZS does not affect other organs and systems, as other *in utero* infections often do.

Some genetic disorders including autosomal recessive disorders caused by pathogenic variants in a number of genes including *JAM3*, *NDE1* ande *ANKLE2* may also cause an abnormal phenotype of foetal brain disruption sequence with somewhat similar neuroimaging abnormalities (Shaheen et al., 2019). Other conditions, such as Aicardi-Goutieres syndrome and pseudo-TORCH syndromes show multiple calcifications but lack other findings seen in CZS (Sanchis et al., 2005; Jean et al., 2020).

One of the main challenges in the diagnosis of infection by ZIKV is laboratory testing. The PCR tests are considered the gold standard for the identification of the Zika virus. However, this type of molecular analysis using viral RNA is only possible in the acute phase of the infection, and up to 80% of adults infected by ZIKV can be asymptomatic or only mildly symptomatic. In the newborn, it is usually difficult to have a positive PCR due to the period between the time of infection *in utero* and diagnostic testing in newborns. Therefore, a negative RT-PCR test does not exclude a congenital infection. Pregnant women with negative viral RNA in blood, urine or amniotic fluid were observed even with proven fetal infection (Baud et al., 2017). Specific serologies can be performed using the ELISA method (Enzyme Linked Immuno Sorbent Assay), with the possibility of detecting immunoglobulin M (IgM) antibodies from 4 to 5 days after infection to 12 weeks after disease. However, there are pitfalls discussed in this methodology, such as the possibility of cross-reacting with other flaviviruses, such as Dengue and Yellow Fever Viruses (Rabe et al., 2016). The plaque reduction neutralization test (PRNT) associated to the ELISA test can improve the sensitivity in dengue-endemic areas (Loyola et al., 2021).

Therefore in the absence of definitive laboratory diagnostic tests, we emphasize the importance of the clinical/epidemiological suspicion of CZS, based on thorough dysmorphologicaland neurological examinations, brain images, and visual evaluations associated in individuals living or travelling to countries where ZIKV is circulating.

### Neurodevelopment in Congenital Zika Syndrome

Supplementary Table S2 Numerous studies consistently showed that 80–100% of the children older than 1 year with CZS have severe forms of cerebral palsy, and they are therefore unable to walk, need to be transported in a manual wheelchair and are limited in their ability to hold their head and trunk and control leg and arm movements (van der Linden et al., 2016a; Alves et al., 2018; Carvalho et al., 2019; Campos et al., 2020; Melo et al., 2020; Pereira et al., 2020; Ventura et al., 2020; Takahasi et al., 2021). The most frequent motor disorder in CZS was spasticity (60–100%), characterized by pyramidal signs, such as hypertonia, hyperreflexia, and persistent primitive reflexes (Carvalho et al., 2019; Pereira et al., 2020; Ventura et al., 2020). The neuromuscular type, characterized by hypotonia, is second in frequency, and the most uncommon type presents dyskinetic signs, dystonia, and/or chorea. Approximately 30% of the children with a neuromuscular clinical presentation also show dyskinetic signs, while a higher percentage of children (40–90%) with a spastic presentation also present dystonic movements and postures during the second year (Pereira et al., 2020; Wheeler et al., 2020). Most children with CZS showed no improvement in motor function and remained relatively stable in their gross motor impairment after the second year (Takahasi et al., 2021).

Approximately one third of children with CZS based on neuroimaging studies and with laboratory confirmation do not present microcephaly at birth, but 87.5% of them develop it postnatally, the degree of microcephaly being severe in most cases. Many of these infants without microcephaly show phenotypic morphological changes in the skull and scalp suggestive of the FBDS (Cavalcante et al., 2021). Even though the majority of these infants with CZS without microcephaly at birth have an unfavorable outcome, one study reported that drug resistant epilepsy, spasticity, and continuous crying were less common among those without microcephaly when compared to those with microcephaly at birth. In addition, the severity of the motor disability was significantly higher in those with microcephaly at birth (Cavalcante et al., 2021).

The functional assessment of the developmental domains of cognition, language and motor skills, with the Bayley Scales of Child Development (BSID) III in these children with such severe motor impairment is difficult and sometimes impossible However, in the few studies that have assessed the development of children with confirmed or suspected CZS using the Bayley Scales of Child Development (BSID) III, all patients showed extremely low-performance scores (<70) in motor, cognitive, and language developmental domains (Carvalho et al., 2019; Marques et al., 2019; Wheeler et al., 2020).

A correlation was observed between more severe alterations in the developmental domains with smaller head size at birth (Carvalho et al., 2019; Melo et al., 2020; Wheeler et al., 2020; Takahasi et al., 2021). Other factors related to the outcome have been poorly studied. Severe reduction in the brain volume and/or malformations of the cortical developmental area in neuroimaging studies (Melo et al., 2020; Takahasi et al., 2021), and the lower economic class (Melo et al., 2020), or poor socioeconomic indicators as well as the occurrence of comorbidities such as dysphagia and epilepsy (Frota et al., 2020; Takahasi et al., 2021), were associated with poorer gross motor skills repertoire. Comorbidities and functional limitations are very common in these children including irritability, continuous crying (>50%) (Cavalcante et al., 2021; Gouvea et al., 2021), difficulty swallowing or dysphagia (>50%) (Frota et al., 2020; Pereira et al., 2020; Gouvea et al., 2021), requiring gastrostomy almost half of the cases (Gouvea et al., 2021), sleep disorders (30–47%), (Campos et al., 2020; Pereira et al., 2020; Gouvea et al., 2021), epilepsy (60–91%) (Sequerra et al., 2020; Cavalcante et al., 2021; Gouvea et al., 2021), and musculoskeletal problems, mainly hip dysplasia (19–43%) are very common (Frota et al., 2020; Pereira et al., 2020). Other comorbidities such as hearing or visual problems have been previously addressed. Although chronic pain is a frequent physical comorbidity of cerebral palsy (CP), only one study has reported no increased pain sensation in patients with CZS using a visual analog scale (Ferreira et al., 2018).

### Neurodevelopment in Exposed Children Without Microcephaly

A major concern following the *in utero* ZIKV exposure is whether the infants born without obvious structural brain abnormalities and/or microcephaly have impairment in development. Only a few studies compared the outcomes of the normocephalic ZIKV-exposed children with those of a neurotypical control group, or with children without ZIKV infection (Supplementary Table S3) (Cardoso et al., 2019; Einspieler et al., 2019; Valdes et al., 2019; Gerzson et al., 2020; Sobral da Silva et al., 2020; Familiar et al., 2021; Grant et al., 2021). Six studies did not find differences between prenatal ZIKV exposure without microcephaly and the unexposed controls (Cardoso et al., 2019; Einspieler et al., 2019; Valdes et al., 2019; Gerzson et al., 2020; Sobral da Silva et al., 2020; Grant et al., 2021). However, one study found altered general movements at 9–20 weeks in 16% of the group exposed to the ZIKV without CZS; abnormal BSID-III in 18% at 12 months; 5% with a score below 70 in at least one domain (Einspieler et al., 2019). In one recent study, ZIKV exposed children showed somewhat lower scores on each subscale of Mullen Scales of Early Learning, except expressive language, but no statistical differences was observed in Fagan test of infant intelligence using eye-tracking measures of fixation and gaze (Familiar et al., 2021).

Studies that have examined the neurodevelopment of children exposed prenatally to ZIKV who do not have a neurotypical group of unexposed infants have shown more controversial and difficult-to-interpret findings. While some studies reported a high percentage of children (24–95%) with the impaired development (Cardoso et al., 2019; Faiçal et al., 2019; Abtibol-Bernardino et al., 2020; Peçanha et al., 2020), these observations have not been consistent in the studies (Cardona-Ospina et al., 2021). The ages at which the children were evaluated were very heterogeneous, as were the neurobehavioral testing approaches. Out of a total of 12 available studies, eight of them used the BSDI-III mainly during the second or third year of life, and the most consistent delays were found in language followed by motor domains (Lopes Moreira et al., 2018; Faiçal et al., 2019; Abtibol-Bernardino et al., 2020; Peçanha et al., 2020; Andrade et al., 2021). Only one study assessed the temporal trajectory of the neurodevelopment in those infants. This study reported an evolving trajectory showing a slow decline from normal scores, mainly in the social cognition and mobility domains as the children became older (Mulkey et al., 2019). A recent study compared the incidence rate of epilepsy among children of ZIKV-infected mothers with those born of uninfected mothers at 1 year of life (Blackmon et al., 2020). This study, using a pediatric epilepsy screening questionnaire and video electroencephalography, detected only two epileptic positive cases in 71 infants whose mothers were ZIKV infected versus 0/71 children born of uninfected mothers, indicating that epilepsy rates could be modestly elevated in children of ZIKV infected mothers.

Despite the availability of numerous studies on the neurodevelopment of children exposed to ZIKV during fetal life, doubts remain about the possible impact on neurodevelopment in the long term. The marked neurodevelopmental difference observed between the studies with a control group and those without a control group is striking. We need more well-designed, studies with matched control-group that examine the long-term neurodevelopmental trajectory, at least until school age, and comorbidities in the children exposed to the ZIKA prenatally who do not have microcephaly and abnormalities on neuroimaging. Evaluations at 5–7 years will be more precise to characterize differences on learning disability and more complex cognitive function. Another challenging point is to reduce the heterogeneity of the assessments, hence the need to use validated and well standardized test that assesses multiple neurodevelopmental domains for different ages, culturally validated and in the native language.

### Estimating Risks of Vertical Transmission and Adverse Outcomes

Only a minority of the ZIKV-exposed pregnancies result in any adverse outcomes. Two different prospective studies in pregnant women with laboratory confirmation of maternal and/or foetal infection referred to tertiary perinatal centers identified the clinical consequences of the gestational infection by the ZIKV but seemingly overestimated the risks of vertical transmission and adverse foetal outcomes. In Rio de Janeiro (Brazil), pregnancy loss, growth deficiency, and birth defects were detected in over 40% of the exposed pregnancies and were higher in the first trimester (55%) and second trimester (52%) than in the third trimester (29%) (Brasil et al., 2016). In French Guyana, documented vertical transmission occurred in 26% of the pregnancies (Pomar et al., 2018). The Zika-associated birth defects occurred in approximately 10% of the babies born to women with the suspected or confirmed recent Zika infection in the U.S. states and approximately 5% babies in the U.S. territories, using both CDC-sponsored Zika pregnancy registries with matched unexposed controls (Reynolds et al., 2017; Shapiro-Mendoza et al., 2017). The percentage of the foetuses or infants with the possible Zika-associated birth defects was estimated at 8, 5, and 4% in the first, second and third trimesters, respectively, in the US registry, and similar risks were identified in Pernambuco and the French territories in the Americas (Honein et al., 2017; de Araújo et al., 2018; Hoen et al., 2018). Complete phenotyping including the quality of the physical examination, was a limitation in all cases. The studies did not use a common set of diagnostic criteria for CZS. For example, in the U.S. Zika Registry, only 25% of cases had neuroimaging and therefore, milder Zika-related brain defects could have been missed (Honein et al., 2017).

Using a Bayesian synthesis in seven prospective studies of the Zika virus in pregnancy with different designs, the vertical transmission was estimated as 47% after maternal infection in the first trimester, 28% in the second trimester, and 25% in the third trimester. The risk of CZS based on at least one of the five diagnostic components was 9% following infections in the first trimester, 3% in the second trimester, and 1% in the third trimester (Ades et al., 2020).

Fatality rates were estimated as 10% (95% CI, 9.2–10.7) in a nationwide linkage study involving 6,059 livebirths of confirmed/possible cases of CZS born in Brazil from 2015 to 2017 (Costa et al., 2020).

## Mechanisms: Lessons From Experimental Studies

### Animal Models and Brain Organoids

Among the animal species are included rodents, non-human primates (Rhesus, Marmoset, Pigtail, Cynomolgus), chicken embryos, porcine, guinea pigs and hamsters (Cugola et al., 2016; Deng et al., 2017; Siddharthan et al., 2017; Pena et al., 2018; Wichgers et al., 2018; Wachholz, 2021). Studies in mice investigated different lineages, different stages of development of the embryo when exposed to ZIKV, different ZIKV strains, different routes of application and dosages; and found a variability of phenotypes including mortality, intra-uterine growth restriction and microcephaly (Yockey et al., 2016; Vermillion et al., 2017; Khaiboullina et al., 2019). Studies in Rhesus monkeys found phenotypes in embryos exposed to ZIKV similar to humans, including smaller brain size, microcalcifications, vasculitis, hemorrhage and apoptosis of neuroprogenitor cells (Adams Waldorf et al., 2016; Martinot et al., 2018). Other non-human primates used to study congenital ZIKV syndrome include marmoset, pigtail and *Cynomolgus* (Pena et al., 2018). Presently, it is clear that the Zika virus is neurotropic and able to infect, disrupt the cell cycle, and trigger cell death of the neural progenitors (Garcez et al., 2016).

Brain organoids, generated from the human pluripotent stem cells, have emerged as a promising technique for modelling the early stages of human neurodevelopment *in vitro*. Experiments using the brain organoids revealed the impact of non-Brazilian and Brazilian Zika strains on the dividing progenitor cells (Qian et al., 2016; Trujillo and Moutri, 2018). It became clear that the virus was preferentially attracted to the dividing neural progenitor cells, including the Integrin αvβ5 complex, due to the presence of a set of exclusively expressed membrane receptors and led to cell death (Vazquez and Jurado, 2020; Wang et al., 2020; Zhu et al., 2020).

Evidence for specific mutations such as the S139N on the viral polyprotein, in the circulating Brazilian ZIKV has emerged (Yuan et al., 2017), but it is unclear whether these variants were responsible for the traumatic phenotypes seen in CZS. The human brain organoid model is also a valuable asset to rapidly repurpose the drugs that could prevent infection (Xu et al., 2016) or block viral replication and eventual vertical transmission (Mesci et al., 2018).

### Immune and Inflammatory Mechanisms

The immune and inflammatory responses in the pathogenesis of the ZIKV are of particular relevance. ZIKV activates AXL signalling, which suppresses the response of the innate immune system (Mlakar et al., 2016). The ZIKV-Human Interactome Map pointed out that the ZIKV infection modulates 27% of the signalling pathways of the immune system (Gurumayum et al., 2018).

Different studies suggest a critical role of pro-inflammatory cytokines in the pathogenesis of infection by ZIKV. Viral transmission from the mother to the foetus occurs through the placenta, which induces the production of interferon type I and pro-inflammatory cytokines, resulting in increased expression of the antiviral genes and neuroinflammation in the brains of newborns (Vermillion et al., 2017). Interferon type I is a possible mediator of the pregnancy complications, such as spontaneous abortions and growth restriction in mice (Lawrence et al., 2019). Expression of the cytokines IL-1β and TNF-α is significantly increased in the neural progenitor cells in mice with ZIKV and human fibroblast cells as an antiviral response to ZIKV (Shao et al., 2016). Other factors with increased levels after ZIKV infection indicate greater recruitment of inflammatory cells, which act to eliminate the virus but exacerbate neuroinflammation (Foo et al., 2018).

In line with this, there is now emerging evidence that both the progression and clinical outcomes of Zika infection are closely associated with glial cells (Greenhalgh et al., 2020; Quincozes-Santos et al., 2021). Glial cells are essential for brain development and homeostasis (Greenhalgh et al., 2020). Glial cells can also participate in neuronal migration, potentially contributing to the development of CZS. Although microglial cells can adopt the inflammatory phenotype to exert neuroprotective functions (Augusto-Oliveira et al., 2021), their persistent activation can be associated with chronic neuroinflammation. This potential event can link Zika infection both to microcephaly and to neurodegenerative/neuropsychiatric diseases in adults (Beys-da-Silva et al., 2020). ZIKV can also affect the immunological and oxidative responses associated with astrocytes (Ledur et al., 2020). Moreover, ZIKV was able to replicate in an experimental model of adult brain tissue (Figueiredo et al., 2019), as well as affected neuron-glia communication in the hippocampus (Bobermin et al., 2020), suggesting that neurological consequences in Zika-infected adults need more studies and clinical observations. By understanding the interaction among neurons and glial cells, preventive/therapeutic strategies may emerge.

### Molecular Markers of Potential Clinical Outcomes

Although research on ZIKV infection is intense, we are still far from understanding the molecular puzzle that results in known clinical phenotypes promoted by the infection. Among those already described outcomes are microcephaly, epilepsy, hearing abnormalities, ocular injury and arthrogryposis (Pierson and Diamond, 2018; Walker et al., 2019). In this sense, many insights on molecular basis of pathological conditions have been made, describing inflammation, immuno-modulation, oxidative stress and signaling pathways implicated in ZIKV-induced neural alterations. These alterations were observed both in vitro and *in vivo* experimental models (Tang et al., 2016; Zhang et al., 2016; Garcez et al., 2017; Hu et al., 2017; McGrath et al., 2017; Tiwari et al., 2017; Nem de Oliveira Souza et al., 2018; Scaturro et al., 2018; Strange et al., 2018; Walker et al., 2019).

High-throughput analyses, such as genomics, transcriptomics, and proteomics, provide insights into the molecular mechanisms and metabolic pathways and can reveal potential targets for the development of new strategies for early diagnosis and therapeutic interventions (Scaturro et al., 2018). Most of the studies integrating systems biology highlight that the neurological phenotypes caused by ZIKV may be associated with 1) downregulation of genes related to neurological development; 2) upregulation of genes related to the inflammatory response; 3) correlation of genes/proteins altered by ZIKV that are also affected in brain malformations and neurological diseases, or 4) upregulation of genes involved with apoptosis (Supplementary Table S4).

**FIGURE 6:**
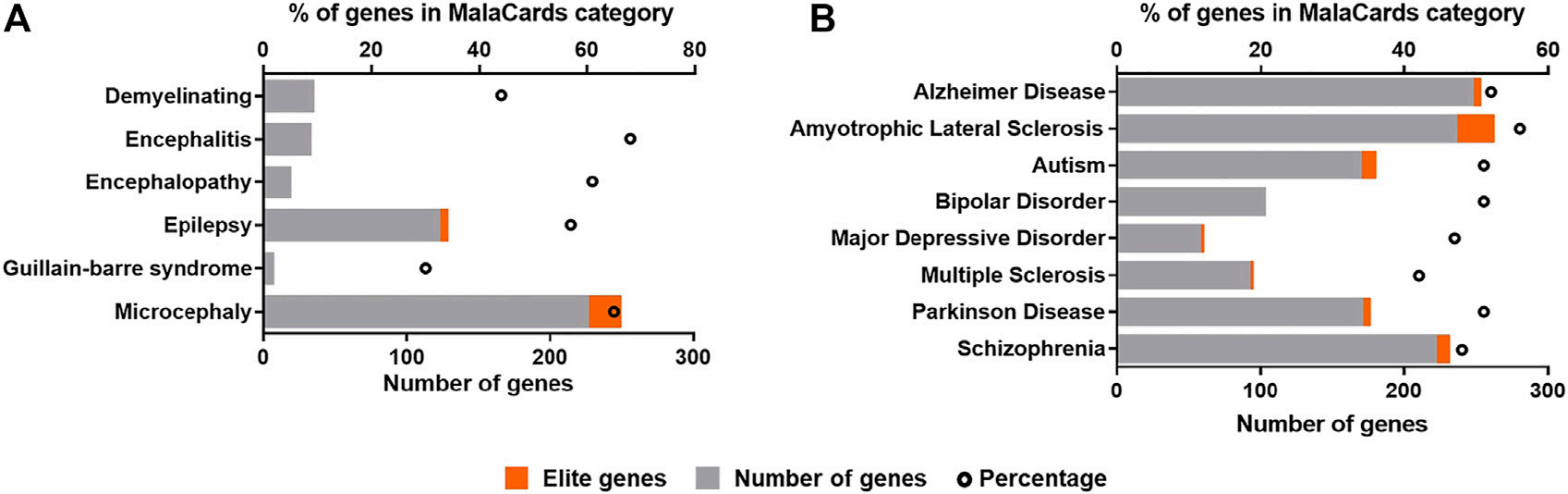
Correlation of differentially expressed genes (DEGs) in ZIKV infection and molecular markers of neurological conditions and brain diseases found in the human disease database MalaCards (https://www.malacards.org). **(A)** Clinical outcomes associated with ZIKV infection. **(B)** Most frequent neurodegenerative and neuropsychiatric diseases in global population. The ZIKV infection DEGs were found in ZIKAVID database (https://zikavid.org). Elite genes are those likely to be associated with causing the disease, according to MalaCards database.

Due to the strong neurotropism of ZIKV, it is clear to assume that the infection may leave footprints, that are being molecularly related to neurological diseases (Adams-Waldorf et al., 2018; Platt et al., 2018; Beys-da-Silva et al., 2019, 2020). Here, we reviewed all differentially expressed genes (DEGs) identified in both *in vivo* and *in vitro* models available up to date in ZIKAVID database (Rosa et al., 2020). A gene is considered differentially expressed if there is a statistically significant difference in expression level between two experimental groups. Further, these DEGs were manually checked for potential assignment to molecular markers of known clinical phenotypes of ZIKV infection, and the most frequent neurodegenerative diseases and neuropsychiatric diseases, as observed in Figure 6.

These DEGs were checked for potential assignment to molecular markers of the already known clinical phenotypes of ZIKV infection available at MalaCards human disease database (Rappaport et al., 2014) (Figure 6A). The same approach was also used for the most frequent neurodegenerative and neuropsychiatric diseases in global population (Figure 6B). This comparison reveals specific molecular markers of ZIKV infection linked to associated pathologies giving further insights of the potential long-term effects of ZIKV infection and risks of clinical consequences in other exposed populations, such as youth and adults, that have been neglected in clinical follow-ups.

Among diseases already associated with ZIKV infection, microcephaly, the first and most dramatic condition detected in ZIKV outbreak in Brazil in 2015, presented the highest number of genes assigned, followed by epilepsy, demyelinating, and encephalitis (Figure 6A). For microcephaly, 21 elite genes out of 248 assigned were found. Elite genes are those likely to be associated with causing the disease, since their gene-disease associations are supported by manually curated and trustworthy sources (Rappaport et al., 2014). Clinical evidences have shown that ZIKV may result in encephalitis and other myelitis, attesting our results (Muñoz et al., 2016; Soares et al., 2016; Da Silva et al., 2017; Pereira et al., 2017; Tolosa et al., 2017; Capasso et al., 2019). As previously observed by Lannuzel et al. (2019), long-term sequelae were frequenting in children and adult patients from ZIKV-outbreak in Guadeloupe and Martinique islands. They identified not only Guillain-Barré syndrome (GBS), but other disorders involving both, central and peripheral nervous system, as encephalitis or encephalomyelitis, in addition to nerve palsies. Interestingly, GBS presented the lowest number of genes assigned. GBS presents only 20 genes identified in MalaCards database, what might explain the low number of markers assigned, corresponding to 30% of all genes for this disease, that have its incidence increased after ZIKV outbreak (Walteros et al., 2019). In this way, the set of molecular markers linked to infection in these associated pathologies can be very helpful to build the specific molecular scenario triggered by ZIKV. Among these markers, we can highlight genes associated with the pathophysiological mechanisms of the CZS and clinical neurological outcomes, as well as common signatures that control the functionality of neural cells, including PTEN, SLC2A1, SLC2A3, SCN1A, MAP2K1, MAP1B, COMT, APP, PPARGC1A, CDKL5, among others.

The number of clinical outcomes and associated pathologies linked to ZIKV infection has been increased rapidly, and still remains to be stablished. According to this, the review of molecular markers of diseases found as DEGs in ZIKV patients and experimental models are important to reveal unknown clinical phenotypes, especially those that might be happening later in life. These molecular clues employed in the seeking for new clinical associations might help to predict some neurological diseases, mainly those difficult of early recognition such as Alzheimer’s disease (AD). The host altered gene expression as consequence of infection might molecularly trigger dramatic diseases, as pinpointed here. Among viral infections, the human immunodeficiency virus (HIV) was already associated to dementia (Major et al., 2000); herpes simplex virus (HSV) may be a trigger for autism (Wadman, 2017), and other human herpesviruses (HV) were previously associated with neurodegenerative diseases, such as AD and multiple sclerosis (Hogestyn et al., 2018). Since ZIKV has become a health problem only after the recent outbreaks few years ago, currently, the lack of information about its long-term effects is considered one of the main concerns (Adams-Waldorf et al., 2018; Beys-da-Silva et al., 2019; Beys-da-Silva et al., 2020; Walker et al., 2019).

The association of ZIKV to other neurological manifestations have also been described and suggested, beyond that observed in congenital Zika syndrome (Lebov et al., 2018; Beys-da-Silva et al., 2019; 2020; Bobermin et al., 2020). Molecularly, according to the review of DEGs in ZIKV infection, other brain diseases can be potentially linked to virus exposure (Figure 6B). Among of them, Schizophrenia and autism presented the highest number of matched genes in the neuropsychiatric category, followed by bipolar disorder and major depressive disorder, all of them assigning 40–50% of all markers for each disease. Autism is one phenotype that was postulated as a potential clinical outcome in ZIKV, due to its molecular impact (Vianna et al., 2018; Beys-da-Silva et al., 2019). Recently a cohort analyzed in an *in utero* exposed children has presented 2.1% of individuals diagnosed with autism (Nielsen-Saines et al., 2019). Although the limitations of this cited report cannot ensure that ZIKV infection is responsible for the disease, our group reported a child diagnosed with autism presented the exposure to ZIKV *in utero* as the only abnormality among all known molecular and genetic causes for autism (Santi et al., 2021). Amyotrophic lateral sclerosis (ALS) and AD have presented an expressive number of genes in the neurodegenerative diseases group, followed by Parkinson’s disease (PD) and multiple sclerosis (Figure 6B). For ALS, elite genes such as *ANG*, *ANXA11* and *ATXN2* have been correlated with familial and sporadic forms of this disease. Moreover, changes in *C9orf72* gene are the most commonly known cause of ALS, and it controls glutamate transporters that are associated with the pathogenesis of the ALS (Fomin et al., 2018). In addition, for autism, one of the elite genes assigned is *PTEN* that can predict deficits in working memory and process new information slowly (Busch et al., 2019). The *APP* and *PLAU* genes, elite genes in MalaCards database for AD, are classically associated with the pathogenesis of AD. *PLAU* is also linked to other aged-related diseases. In this sense, ZIKV may accelerate the progression of cognitive decline in adult or elderly population. Concerning Parkinson’s disease (PD) elite genes assigned to ZIKV infection DEGs, such as *ATXN2*, *GBA*, *MAPT*, and *SNCAIP*, might synergistically act to influence the onset of this disease after ZIKV exposure (Pascale et al., 2016; Beys-da-Silva et al., 2020). It is important to note that the total number of genes can change several neurochemical parameters that will be able to contribute to other clinical symptoms associated with neurodegenerative/neuropsychiatric diseases. In this context, schizophrenia that is a complex disorder involving dysregulation of multiple pathways in its pathophysiology showed *COMT* as an elite gene, as well as *ACHE*, *C9orf72*, and *BDNF* in correlated genes, and all of them are crucial for neurotransmitter systems, which are strongly altered in this disease. A more expressive number of genes, including elite genes, were found in pathologies not yet associated. Not surprisingly, ZIKV infection has being associated to other complex diseases, such as dementia, PD, and schizophrenia in different models and reports (Adams-Waldorf et al., 2018; Platt et al., 2018; Beys-da-Silva et al., 2019; Beys-da-Silva et al., 2020). Recently, it was suggested that ZIKV exposure could lead to severe neuropathological complications in patients with preexisting condition, such as multiple sclerosis (Alves-Leon et al., 2019). However, these consequences could be caused directly by virus or host immune response, and this need to be further evaluated (Souza et al., 2019).

The alert on the molecular markers and potential pathologies above, make clear the relevance on monitoring, not only those infants exposed *in utero*, but other groups infected by the virus, such as children, adults and the elderly. This reinforces the importance of maintaining the surveillance of individuals ZIKV-exposed for long-term outcomes. Therefore, in view of the molecular markers pinpointed and potential association of ZIKV infection with diseases that may appear in future, the review of these data not only indicates the relevance of clinical follow-up of these patients, but also lists genes that could be analyzed in specific cohorts.

### Cofactors in CZS Development

It is still a question of how CZS is modulated by various cofactors, from genetic predisposition to environmental difference, that could interfere with multiple stages of the infection process (Barbeito-Andrés et al., 2018; Caires-Júnior et al., 2018). Neural stem cells derived from the iPSCs from discordant affected dizygotic twins showed that ZIKV replicates significantly more in the cell culture of the microcephalic patients, and their genetic profile revealed that differential susceptibility to ZIKV is related to multiple genes or epigenetic mechanisms (Caires-Júnior et al., 2018).

Similarly, the environmental elements could enhance the ZIKV neurotoxicity. The highest prevalence of CZS is in Northeast Brazil, where seasonal drought periods favors harmful cyanobacteria blooms (de Oliveira et al., 2017). Saxitoxin, a toxin produced by cyanobacteria, has been shown to enhance the cell death associated with ZIKV in both brain organoids and mice (Pedrosa et al., 2020). Additionally, the Northeast region has higher rates of malnutrition than other regions of Brazil (de Oliveira et al., 2017; Barbeito-Andrés et al., 2020). Protein malnutrition is known to be critical for the maturation of the immune cells. Immunocompetent pregnant mice fed with protein restriction when infected showed a significantly higher load of ZIKV in comparison to controls, enhanced placental damage, and microcephalic offspring. Folic acid has been shown to prevent placental and brain injuries caused by ZIKV in human cells and a mouse model (Simanjuntak et al., 2020).

Moreover, epidemiological data showed that poverty is correlated with the incidence of microcephaly associated with ZIKV, and this involves a lack of proper sanitation, water hoarding by poorer families leading to the spread of the mosquito vector, working outdoors, demanding access to health care, and poor nutrition (Caires-Júnior et al., 2018).

## Concluding Remarks

As of July 2019, a total of 87 countries and territories had evidence of autochthonous mosquito-borne transmission of Zika virus (ZIKV), and 31 countries reported cases of microcephaly/CZS. The incidence of ZIKV infection in the Americas peaked in 2016 and declined thereafter. The Pan American Health Organization (PAHO/WHO) counted 864,543 cumulative cases from 2015–2020 in the Americas region, and Brazil had the highest number of cases (Bailey and Ventura, 2018). There is no vaccine available to prevent ZIKV infection or effective antiviral treatment. A review in the Cochrane Library database performed in March 2021 identified 22 trials for the Zika vaccine, but none are commercially available yet (Cunha et al., 2020). Therefore, preventive measures at the community level, such as individual measures, are the key to reducing its impact.

CZS encompasses a broad range of sensorimotor impairments with multiple health and social effects. Therefore, comprehensive care for the child and family requires multidisciplinary involvement in undertaking the holistic needs assessment. Many of the therapies are the same as those for cerebral palsy, and it is imperative to engage and support families/caregivers of children with CZS who are chronically disabled, requiring multifaceted treatments. Multisensory integration of vision-specific proprioceptive stimuli and auditory, motor, and cognitive functions plays an essential role in improving child’s skills and quality of life. They should be included in a psychosocial group, and a strategy should be implemented to improve the caregivers’ psychosocial and physical health (Diniz et al., 2020).

Different organizations have published good guidelines for the treatment and care of children with CZS (Adebanjo et al., 2017). However, we should take into account that the majority of the countries with the highest prevalence of CZS are those with the highest social disparities and poverty rates. In Brazil, as in other Latin American countries, the lack of information and poor access to family planning associated with the criminalization of abortion even in cases of congenital malformations contributed to a disproportionately heavier burden on more impoverished families and geographic asymmetries in birth prevalences of CZS (Cunha et al., 2020; Diniz et al., 2020). The same inequality is also reflected in the access to care for the affected families. Northeast Brazil had the highest number of affected children with a shortage of specialized professionals, mostly concentrated in the capital cities (Peiter et al., 2020). The distances between the families’ residence to the health care providers often create difficult displacements associated with physical and mental distress. The burden for the family of caring for an affected child is also financial since it can even cause the inability to work of the mother/caregiver and the loss of a job and has heavy consequences on their mental health and quality of life (Peiter et al., 2020).

In rehabilitation, the International Classification of Functioning, Disability and Health (ICF) includes perceptions of parents and caregivers regarding the issues for their children with CZS. Parents/caregivers highlighted the importance of environmental factors and motor function for their children with CZS. The use of the ICF as a guide for this therapeutic planning enables a holistic framework for considering perspectives that extend beyond the issues with body functions and structures for children with CZS (Campos et al., 2020). Similarly, some researchers have stressed the importance of considering CZS in the framework of a “social model of disability”, changing the focus from the biomedical-only approach to include deeper perspectives from the families involved, building more horizontal relationships between the health services and patients/families and stronger involvement from governments (Mendes et al., 2020).

Finally, our review encompasses 6 years of follow-up from the first case reported in the literature, and the possible behavioral and cognitive development of the children presently classified as unaffected or mildly affected is still unknown.
