# Supplementary material for: Neurodevelopment in Children Exposed to Zika in utero: Clinical and Molecular Aspects
Source: Front Genet. 2022 Mar 8;13:758715. doi: 10.3389/fgene.2022.758715 (PMC8957982; doi:10.3389/fgene.2022.758715)
Supplement: Supplementary file 1 [file Table1.docx]

| **Supplemental Table 1. Visual and hearing outcomes of children with Congenital Zika Syndrome (CZS) with or without microcephaly** | | | | | | |
| --- | --- | --- | --- | --- | --- | --- |
| **Visual outcomes** | | | | | | |
| **Study design/sample size (n)/reference** | **Age range (months)** | **Type of diagnosis** | **Geographical area** | **Main neurologic outcomes (%)** | **Main structural ocular abnormalities (%)** | **Main visual function outcomes (%)** |
| Cross-sectional, case series: Suspected CZS (10 infants; 20 eyes) / Ventura et al., 2016 | 0.7–2.9 | Clinical findings of CZS | Pernambuco state/ northeast of Brazil | Microcephaly (100) | Structural^b^ in 17/20 eyes (85); retinal^c^ in 15/20 eyes (75); ON^d^ in 4/20 eyes (45) | Strabismus in 6/10 infants (60); nystagmus in 1/10 infants (10) |
| Case-series: Suspected CZS (29 infants; 17 eyes) / de Paula Freitas et al., 2016 | 1–6 | Clinical findings of CZS | Bahia state/ northeast of Brazil | Microcephaly (100) | Structural^b^ in 17/58 eyes (29); retinal in 11/17 eyes (65); ON in 8/17 eyes (47); bilateral iris coloboma in 2/17 eyes (12); lens subluxation in 1/17 eyes (6) |  |
| Cross-sectional: Confirmed CZS (32 infants; 64 eyes) / Ventura et al., 2017a | 4–7 | Laboratory testing, infant (MAC-ELISA on CSF) | Pernambuco state/ northeast of Brazil | Microcephaly (100); Seizure (69); brain calcifications (97); cerebellar/brainstem hypoplasia (38); hypoplastic corpus callosum (88). | Retinal findings in 18/64 eyes (28); ON in 11/64 eyes(17); retinal vessels attenuation in 2/64 eyes (3); straightening retinal vessels in 2/64 eyes (3) | Abnormal binocular visual acuity in 22/30 infants (73); abnormal visual development in 31/32 infants (97); strabismus in 24/32 infants (75); nystagmus in 9/32 infants (28); reduced contrast sensitivity in 20/31 infants (65); hypo-accomodation in 5/14 infants (36) |
| Prospective, case-series: Suspected CZS (43 infants) / Yepez et al., 2017 | 0.2–6.6 | Clinical findings of CZS | Colombia and Venezuela | Microcephaly (100) | Ocular findings in 43 infants (100); Anterior segment (12); structural^b^ (88);  ON^d^ in 5 infants (12); pigment mottling in 27 infants (63); lacunar maculopathy in 3 infants (7); chorioretinal scarring in 3 infants (7); combination of lesions in the posterior pole in 11 infants (26); congenital glaucoma in 5 infants (12). |  |
| Cross-sectional: Suspected CZS (70) / Verçosa et al., 2017 | 1–8 | Clinical findings of CZS | Ceará state/ northeast of Brazil | Microcephaly (100) | Ocular findings in 25 infants (36); structural^b^ in 18 infants (26); retinal in 15 infants (21) in 27 eyes (19); ON in 10 infants (14) in 17 eyes (12%) | Strabismus or nystagmus in 7 infants (10) |
| Case-series, a cohort: ZIKV exposed in-utero (112) / Zin et al., 2017 | 0–10.2 | Laboratory testing; mother (RT-PCR) | Rio de Janeiro state/ southeast of Brazil | Microcephaly in 20 infants (18); other CNS abnormalities^a^ in 31 infants (28); no CNS findings in 61 infants (55) | Structural^b^ in 24 infants (21); retinal^c^ in 15 infants (13); ON^d^ in 19 infants (79); microphthalmia in 1/112 infants (4) | Nystagmus in 6/24 infants (25) |
| Cross-sectional, case control: Confirmed CZS (119) / Ventura et al., 2018 | 6–13 | Laboratory testing, infant (MAC-ELISA on CSF) | Pernambuco state/ northeast of Brazil | Microcephaly in 100/113 infants (89); severe microcephaly in 73/100 infants (73) | Retinal^c^ in 74/234 eyes (32); ON^d^ in 63/117 eyes (26.9) | Abnormal binocular visual acuity in 107 infants (90); abnormal visual development milestones in 100/108 infants (93); strabismus in 95/119 infants (80); nystagmus in 54/119 infants (45); reduced contrast sensitivity in 87/107 infants (81); visual field defect in 41/91 infants (45) |
| Cross-sectional: Confirmed CZS (60) / Ventura et al., 2017b | 9.0–16.0 | Laboratory testing, infant (MAC-ELISA on CSF) | Pernambuco state/ northeast of Brazil | Microcephaly in 51/60 children (85); mild microcephaly in 14/51 children (28); severe microcephaly in 37/51 children (73) | Structural^b^ in 48/115 eyes (42) of 30/58 children (52); Retinal^c^ in 35/115 eyes (30); ON^d^ in 26/115 eyes (23); vessels attenuation in 2/115 eyes (2); cataract, microcornea, microphthalmia in 1 children (2); significant refractive error in 52/119 eyes (44%) in 29/60 children (48) | Abnormal binocular visual acuity in 60 children (100); hypo-accommodation in 17/21 children (81); strabismus in 55/60 children (92); nystagmus in 28/60 children (47) |
| Prospective cohort, Cross-sectional: Suspected or Confirmed CZS (224) / Tsui et al., 2018 | 0.4–3.3 | Laboratory testing, mother (RT-PCR); Clinical suspicion; mother; fetal ultrasound findings; Laboratory testing, infant (RT-PCR); Clinical findings of CZS | Rio de Janeiro state/ southeast of Brazil | Microcephaly in 62/224 infants (28); other CNS abnormalities and microcephaly in 90/224 infants (40) | Eye abnormalities in 57/224 infants (25); retinal^c^ in 37/224 infants (17); ON^d^ in 44/224 infants (20); unilateral microcornea, inferior iris coloboma, and optic nerve coloboma in 1/224 infant (0.4); unilateral microcornea and microphthalmia in 1/224 infant (0.4); ON atrophy, retinal vessel attenuation, and macular chorioretinal scar in 1/224 infant (0.4) |  |
| Retrospective study: Confirmed CZS (70) / Campos et al., 2020 | 0–15.5 | Laboratory testing, infant (MAC-ELISA on CSF) | Pernambuco state/ northeast of Brazil | Neuroimaging: occipital volume loss (95), ON atrophy (12), chiasmal atrophy (4), globe abnormality (1.4) | Structural ocular abnormality in 34/62 infants (55): fundus findings in 34 infants (55), optic nerve findings in 24 infants (39). No anterior segment findings. | Visual impairment identified in 25/25 infants (100): mild in 2 infants (7), moderate in 5 infants (19), severe in 7 infants (27), profound in 3 infants (12), near blindness in 7 infants (27), blindess in 1 infant (4). |
| Retrospective: CZS (469) / Ventura et al., 2021 | 0–36 | Clinical manifestations of CZS  and positive reverse transcription polymerase-chain-reaction (RT-PCR) and/or serology  for the Zika virus | Rio de Janeiro state / southeast of Brazil;  Pernambuco and Bahia states / northeast of Brazil | Microcephaly in 214 children; 62 cases were severe | Ocular manifestations were found in 269 of 938 eyes (28.7%; 148/469 children [31.6%]). The main ocular alterations were optic nerve pallor in 122 of 938 eyes (13.0%), focal pigment mottling in 112 eyes (11.9%), and chorioretinal scars in 101 eyes (10.8%) |  |
| **Hearing outcomes** | | | | | | |
| **Study design/sample size (n)/reference** | **Age range (months)** | **Type of diagnosis** | **Geographical Area** | **Main Neurologic outcomes (%)** | **Audiological evaluation (methodology)** | **Main audiological outcomes (%)** |
| Prospective observacional study (78) / Faria et al., 2020 | 0.75–3.25 | Group 1: Laboratory testing, mother (RT-PCR) and Clinical findings of CZS  Group 2: (control) asymptomatic children; negative mothers  Group3: Clinical findings of CZS; mothers with exanthema without laboratory confirmation  Group 4: Clinical findings of CZS; mothes without exanthema without laboratory confirmation | Rio de Janeiro State/ southeast of Brazil | CZS abnormalities:  Group1: 9/36  Group2: 0/24  Group3: 12/12  Group4: 6/6 | NHS: aABR : (test/retest)  Diagnosis: ABR specific frequency with 500 and 2000 Hz tone burst CE-Chirp stimuli in air and bone pathway. | NHS: test: 6/78 (7.7); retest: 4/78 (5,1) “FAIL”  Diagnosis: 3 children (group 3 and4) with sensorineural hearing loss (3.8) |
| Cross-sectional: CZS (70) / Leal et al., 2016 | 0–10 | Laboratory testing, infant (MAC-ELISA on CSF) | Pernambuco State/ northeast of Brazil | Microcephaly in all children; severe microcephaly in 43/70 children | NHS: a ABR : ( test/ retest)  Diagnosis: ABR specific frequency with 500 and 2000 Hz tone burst stimuli in air and bone pathway. | NHS: test: 16/70 (22); retest: 8/70 (11.4) “FAIL”  Diagnosis: 4 sensorineural hearing loss (5.8) |
| Cross- sectional: ZIKV exposed *in-utero* (43) / Fandiño-Cárdenas et al., 2019 | 3–24 | Epidemiological and clinical criteria based on maternal symptoms (ZIKV-exposed infants group) | Cesar, Colombia | ZIKV-exposed infants: 3 with microcephaly | NHS: 1 test DPOAEs; retest: tympanogram and aABRs at 35 dBnHL  Follow up (24 month): DPOAEs. | NHS: test: Zika-exposed: 10/43 (23.2); Control: 4/23 (17.4); Retest: 0/10 (0) “FAIL”  Follow up 24m: Zika-exposed: 31/43; Control: 12/23  No sensorioneural hearing loss |
| **Abreviations:** Visual: MAC-ELISA (antibody-capture enzyme-linked immunosorbent assay); CSF (cerebrospinal fluid); RT-PCR (reverse transcription - polymerase chain reaction); CNS (central nervous system); CZS (congenital Zika syndrome); ON (optic nerve). Auditory: NHS (newborn hearing screening); aABR (auditory brainstem reponse); DPOAEs (distortion product otoacoustic emissions)  ^a^Other CNS abnormalities includes ventriculomegaly, cerebral calcifications, posterior fossa abnormalities, pachygyria, and lissencephaly.  ^b^ Structural abnormalities includes retinal and optic nerve findings.  ^c^Retinal includes pigment mottling and chorioretinal atrophy,  ^d^ Optic Nerve includes hypoplasia, pallor, increased optic cup. | | | | | | |

**References**

Campos, T., Schiariti, V., Gladstone, M., Melo, A., Tavares, J. S., Magalhães, et al. (2020). How congenital Zika virus impacted my child's functioning and disability: a Brazilian qualitative study guided by the ICF. *BMJ open*, *10*(12), e038228. doi: 10.1136/bmjopen-2020-038228

de Paula Freitas, B., de Oliveira Dias, J. R., Prazeres, J., Sacramento, G. A., Ko, A. I., Maia, M., et al. (2016). Ocular Findings in Infants With Microcephaly Associated With Presumed Zika Virus Congenital Infection in Salvador, Brazil. *JAMA ophthalmology*, *134*(5), 529–535. doi: 10.1001/jamaophthalmol.2016.0267

Fandiño-Cárdenas, M., Idrovo, A. J., Velandia, R., Molina-Franky, J., & Alvarado-Socarras, J. L. (2019). Zika Virus Infection during Pregnancy and Sensorineural Hearing Loss among Children at 3 and 24 Months Post-Partum. *Journal of tropical pediatrics*, *65*(4), 328–335. doi: 10.1093/tropej/fmy055

Faria, A., Miterhof, M., Vianna, R., Carvalho, F. R., Dalcastel, L., Oliveira, S. A., et al. (2020). Audiological Findings in Children Suspected to Have Been Exposed to the Zika Virus in the Intrauterine Period. *Otology & neurotology*, *41*(7), e848–e853. doi: 10.1097/MAO.0000000000002704

Leal, M. C., Muniz, L. F., Ferreira, T. S., Santos, C. M., Almeida, L. C., Van Der Linden, V., et al. (2016). Hearing Loss in Infants with Microcephaly and Evidence of Congenital Zika Virus Infection - Brazil, November 2015-May 2016. *MMWR. Morbidity and mortality weekly report*, *65*(34), 917–919. doi: 10.15585/mmwr.mm6534e3

Tsui, I., Moreira, M., Rossetto, J. D., Vasconcelos, Z., Gaw, S. L., Neves, et al. (2018). Eye Findings in Infants With Suspected or Confirmed Antenatal Zika Virus Exposure. *Pediatrics*, *142*(4), e20181104. doi: 10.1542/peds.2018-1104

Ventura, C. V., Maia, M., Bravo-Filho, V., Góis, A. L., & Belfort, R., Jr (2016). Zika virus in Brazil and macular atrophy in a child with microcephaly. *Lancet*, *387*(10015), 228. doi: 10.1016/S0140-6736(16)00006-4

Ventura, C. V., Zin, A., Paula Freitas, B., Ventura, L. O., Rocha, C., Costa, F., Nery, N., Jr, De Senna, T., Lopes Moreira, M. E., Maia, M., & Belfort, R., Jr (2021). Ophthalmological manifestations in congenital Zika syndrome in 469 Brazilian children. *Journal of AAPOS : the official publication of the American Association for Pediatric Ophthalmology and Strabismus*, 25(3), 158.e1–158.e8. do: 10.1016/j.jaapos.2021.01.009

Ventura, L. O., Lawrence, L., Ventura, C. V., Dutton, G. N., Marinho, P., Ferro, et al. (2017). Response to correction of refractive errors and hypoaccommodation in children with congenital Zika syndrome. *Journal of AAPOS*, *21*(6), 480–484.e1. doi: 10.1016/j.jaapos.2017.07.206

Ventura, L. O., Ventura, C. V., Dias, N. C., Vilar, I. G., Gois, A. L., Arantes, T. E., et al. (2018). Visual impairment evaluation in 119 children with congenital Zika syndrome. *Journal of AAPOS*, *22*(3), 218–222.e1. doi: 10.1016/j.jaapos.2018.01.009

Ventura, L. O., Ventura, C. V., Lawrence, L., van der Linden, V., van der Linden, A., Gois, A. L., et al. (2017). Visual impairment in children with congenital Zika syndrome. *Journal of AAPOS*, *21*(4), 295–299.e2. doi: 10.1016/j.jaapos.2017.04.003

Verçosa, I., Carneiro, P., Verçosa, R., Girão, R., Ribeiro, E. M., Pessoa, A., et al. (2017). The visual system in infants with microcephaly related to presumed congenital Zika syndrome. *Journal of AAPOS*, *21*(4), 300–304.e1. doi: 10.1016/j.jaapos.2017.05.024

Yepez, J. B., Murati, F. A., Pettito, M., Peñaranda, C. F., de Yepez, J., Maestre, G., et al. (2017). Ophthalmic Manifestations of Congenital Zika Syndrome in Colombia and Venezuela. *JAMA ophthalmology*, *135*(5), 440–445. doi: 10.1001/jamaophthalmol.2017.0561

Zin, A. A., Tsui, I., Rossetto, J., Vasconcelos, Z., Adachi, K., Valderramos, S., et al. (2017). Screening Criteria for Ophthalmic Manifestations of Congenital Zika Virus Infection. *JAMA pediatrics*, *171*(9), 847–854. doi: 10.1001/jamapediatrics.2017.1474
