# Supplementary material for: Neurodevelopment in Children Exposed to Zika in utero: Clinical and Molecular Aspects
Source: Front Genet. 2022 Mar 8;13:758715. doi: 10.3389/fgene.2022.758715 (PMC8957982; doi:10.3389/fgene.2022.758715)
Supplement: Supplementary file 2 [file Table2.docx]

| **Supplemental Table 2. Development of children with Congenital Zika Syndrome (CZS)** | | | | | |
| --- | --- | --- | --- | --- | --- |
| **Type of Study / sample size (n) / reference** | **CZS Sample size** | **Zika laboratory confirmation** | **Age of evaluation*** | **Assessment** | **Main Outcomes** |
| Case-series. CZS with normal head size. Van den Linden et al., (2016) | 13 | Yes | 5-12m | Neuro exam | All (100%) motor impairment.  Comorbidities: dysphagia 11/13 (77%), epilepsy 7/13 (54%), 3/13 (23%) chorioretinal abnormalities, 2/13 abnormal hearing evaluation. Postnatal development of microcephaly 11/13 (77%). |
| Cross-sectional  Satterfield-Nash *et al*., (2017) | 19 | Yes | 19-24m | HINE,  ASQ-3 | 15 (79%) severe motor impairment. 15 patients (79%) did not pass the ASQ-3 age interval questionnaire designed for a child aged 6 months.  Comorbidities: sleeping disturbances, 9 (47%) feeding difficulties, 11 (58%) vision and 13 (68%) hearing abnormalities. |
| Cross-sectional  França *et al*., (2018) | 8 | No  (clinical criteria) | 21±2m | BSID-I | All extremely low performance in the motor (47±2) and cognitive (55±0) development domains. |
| Case-series  Alves *et al*., (2018) | 24 | No  (clinical criteria) | 16-24m | DDST II | All (100%) severe delay in language, motor (gross, fine/adaptative) and personal/social domains. Epilepsy 23 (96%). |
| Cross-sectional  [Ferreira](https://www.ncbi.nlm.nih.gov/pubmed/?term=Ferreira%20HNC%5BAuthor%5D&cauthor=true&cauthor_uid=29844290) *et al*., (2018) | 34 | No  (clinical criteria) | 21±7m | ICF  & ICF Core Set for cerebral palsy | All (100%) severe disability of mental functions of language and >80% severe or complete disability in motor and intellectual functions as well as personal interactions 95% extreme difficulty in performing age-appropriate fine hand movements. They were unable to use any type of utensils or drinking vessels.  Comorbidities: altered sleep 20.6%, sensation of pain 11.8%. |
| Cross-sectional  Wheeler *et al*., (2018) | 47 | Yes | 16±3m | ASQ-3  BISQ | All (100%) well below developmental expectations. Sleep problems in 18%. |
| Case-series  Marques *et al*., (2019) | 39 | Yes 24 (61.5%)  No (clinical criteria)  15 (38.5%) | 6-18m | AIMS  BSID-III | All (100%) marked delay in gross motor development. At the age of 18 months, the mean AIMS corresponding to a motor development equivalent to 4 to 5 months of age. Gross motor function score in BSID III at 12 m was 10.76±11.77. Cerebral palsy: 35 (90%). |
| Case-series: CZS with cerebral palsy  Carvalho *et al*., (2019) | 82 | No (clinical criteria) | 13±2.1m | BSID-III  GMFCS | 96% Spastic cerebral palsy; GMFCS level IV-V 86%, absent expected postural reactions 93%, persistence of primitive reflexes 95%. Epilepsy 63%. Extremely low performances on cognitive (95%), language (98%) and motor (98%) developmental scores. Positive correlation between the cognitive score and birth head circumference. Abnormalities in evoked visual potentials 7% and brainstem auditory evoked potentials 13%. |
| Case-series. CZS with cerebral palsy  Carvalho *et al*., (2019) | 69 | No  (clinical criteria) | Median 24m (23-32) | BSDI-III  GMFCS | All (100%) spastic cerebral palsy; quadriplegic 91%, hemiplegic 6%, diplegic 3%. GMFCS level IV-V in 93% of the patients. Epilepsy 73%. Practically all had extremely low performances in each domain: cognitive (57.0±7.6), language (49.4±6.9) and motor (47.8±6.7). |
| Case-series  Lima *et al*., (2019) | 16 | No  (clinical criteria) | 6-24m | PEDI | Delay in the domains of self-care, social function and mobility.  Comorbidities: ophthalmologic alterations 69%, hearing alterations 19%, seizures 75% and gastroesophageal reflux 25%. |
| Case-series  Vianna *et al*., (2019) | 26 | Yes | 18m | DDST II | All (100%) motor abnormalities and developmental delay. Epilepsy 69%.  Comorbidities: dysphagia 92%. Ophthalmologic disorders 46% (optic nerve hypoplasia, macular scarring, pigmentary retinal mottling, coloboma). |
| Cohort study.  35 CZS, 76 prenatally exposed to ZKV without CZS, and 333 control group.  Einspieler *et al*., (2019) | 35 | No  (clinical criteria) | 9-20 wk  12 mo | General movements assessment  MOS  BSID-III | CZS cohort. Altered general movements 100% at 9-20 weeks of age and all scored in the lowest range of the MOS and its 5 subscales. All (100%) had cerebral palsy with GMFCS level V. Epilepsy 79.4%. |
| Case-series  Wheeler et al., (2020) | 121 | Yes | 31±1.9m | BSID-III | 97.5% severe developmental delays in all domains of functioning; mean developmental age equivalent to approximately 2 to 4 months. Cognitive domain: 55.3 ±1.8 (range 55-70). Motor domain: 46.3 ±1.83 (range 46-64). Language domain: 47.7. ±2.89 (range 47-71). Receptive language age-equivalent scores were significantly higher than most other domains. Head circumference at birth was the single strongest factor associated with outcomes across all developmental domains. |
| Case-series  Melo *et al*., (2020) | 59 | Yes 27  No (clinical criteria) 32 | 14.7±3.9m  (5-29) | GMFM-66 GMFCS | All (100%) cerebral palsy; 93% GMFCS level IV-V. Severe malformations of cortex and small head circumference at birth were predictors of lower scores. |
| Case-series  Ventura *et al*. (2020) | 77 | No  (clinical criteria) | 24m | GMFM-66  GMFCS | Cerebral palsy (100%), 96% with GMFCS level V: quadriplegia 95%. Neuroimaging of children with hemiplegia and diplegia showed asymmetric and localized lesions, respectively, whereas children with quadriplegia presented diffuse lesions and severe malformations of cortical development. |
| Cohort study.  Bertolli et al., (2020) | 43 | Yes 43 (anthropometric and laboratory criteria).  77 only lab criteria or anthropometric criteria | Median age 23m  (19-26) | HINE  ASQ3 | Infants with anthropometric and laboratory criteria: severe motor impairment (61%), cerebral palsy (58%). Developmental delay (79%). Impaired response to auditory and visual stimuli: 54% and 49, respectively. Ocular abnormalities: retinal (16%); abnormal fixation and following (37%). Seizure screening positive (43%).  Infants with only laboratory criteria or anthropometric criteria: Developmental delay mild/moderate (46%); severe (5%). |
| Cross sectional  Souza Pereira et al., (2020) | 75 | Yes 15  No (Clinical criteria) 60.  (All recruited infants had neuroimaging anomalies) | Median age 33m  (26-40 IQR) | GMA  GMFM66  MACS  EADCS  CFCS  GMFCS | According to the clinical picture, children divided in: corticospinal group (predominance of pyramidal signs) 48 (64%), neuromuscular group (predominance of hypotonia) 23 (31%) and a group with only dyscinetic signs 4 (5%). However, dyskinesia was associated in 19/48 (39.6%) in the corticospinal group and in 7/23 (30.4%) in the neuromuscular group.  Cerebral palsy GMFCS Level 5 in 80% of corticospinal group and in 100% of neuromuscular group. All functional scales showed a predominance of severe impairment.  Comorbidities: 69% Epilepsy, 60% dysphagia; 37% sleep disorders, 43% hip dislocation. Hearing affected in 11% and vision in 39%. Pneumonia 47%, urinary tract infection 44%. |
| Cross-Sectional.  -Group 1: Severe  microcephaly 114;  - Group 2 moderate microcephaly 20;  -Group 3 prenatal ZIKV no microcephaly;  -Group 4 control-group 46.  Sobral da Silva et al., (2020) | 134 | Yes | 10-45m | SWYC  BPSC  PPSC | Group 1. Significant higher frequency of abnormal neurological findings (98.2% vs) and neuroradiological (97%) alterations than the other groups. Pyramidal syndrome 89.2%, inadequate responses to visual stimuli 57%, and inadequate responses to auditory stimuli14%.  The frequency of seizures differed across the ZIKV-exposed groups: a 67.9% in Group 1, 17.6% in Group 2, and 2.2% in Group 3. None of the children in Group 1 were able to perform the milestone skills expected for their chronological age. For Group 2, SWYC screening indicated risks of developmental delay in 65% of the children.  Group 3 and Group 4 had similar frequencies of children at risk of development delay. |
| Case-Series: CZS with cerebral palsy  Takahasi et al., (2020) | 100 | Yes 36  No (clinical criteria) 64 | 25.6 ± 5.5m | GMFCS  GMFM-88  GMFM-66 | 89% severe cerebral palsy (GMFCS level V). Low economic class, microcephaly at birth, epilepsy, and brain parenchymal volume loss associated with low GMFM-66 scores. |
| Case-Series  Da costa Pinheiro Frota et al., (2020) | 50. | No  (clinical criteria) | 24 m | GMFCS  GMFM-88 | 100% cerebral palsy: GMFCS level V 71.7%, level IV 23.9% and levels I or II 4.3%.  Comorbidities. Dysphagia (90%), visual impairment (58%), epilepsy (48%), hip dislocation (20%) and arthrogryposis (14%).  The combination of dysphagia and epilepsy in children with CZS was associated with worst gross motor function. Head circumference was not associated with poorer gross motor skills repertoire. |
| Case Series.  García-Boyano, (2020) | 16 CZS  5 no CZS | Yes | 23.6±4.5m  3 not follow (2 died and  1 lost) | Haizea–Llevant screening test | 100% neurodevelopmental delay, spasticity and hyperreflexia.  Comorbidities. Most of them (14/15) recurrent epileptic seizures (12 epileptic spasms) and dysphagia (12/13). Abnormal visual evoked potential and EABR responses abnormal in 50% and 37.5%, respectively of CZS cases. Hip dysplasia 19%.  5 cases without CZS. Not clinical impairment at 23 months of age. All were considered normal during follow up until 23 months. In 4 with neuroimaging studies: normal. |
| Qualitative study  Correia Campos, 2020 | 32 | Yes (number not reported)  No (clinical criteria) | 0-4 y  32.5 ±6.2 | Qualitative study. GMFCS | All (100%) severe motor impairment (all GMFCS level V).  Comorbidities: Vision problems 69%, Hearing 31%**,** Sleep problems 31% and Epilepsy 34%. |
| Prospective cohort study.  Cavalcante, 2021 | 93 | Yes | Up to 36 m | GMFCS  Ocular examination | Sixty-one cases (66%) presented with microcephaly at birth whereas 32 (34%) were born without microcephaly. Maternal ZIKV infection was most frequently reported in the first trimester of pregnancy among those born with microcephaly (79.2%) compared with those born without microcephaly (46.1%).  Spasticity (97%), epileptic seizures (90.7%). Difficulty sleeping, irritability, continuous crying, and dysphagia were present in most cases with or without microcephaly. However, drug resistant epilepsy, spasticity, and continuous crying were less common among those without microcephaly. In addition, the severity of the motor disability was significantly higher in those with microcephaly at birth. Furthermore, any ophthalmic damage and bilateral retina damage were also more frequently among those with microcephaly at birth. |
| Age of evaluation: mean ± standard deviation or mediam (range). m=months, wks = weeks, y = years. AIMS - *Alberta Infant Motor Scale;* ICF: *The International Classification of Functioning Disability and Health*; ASQ-3 *Ages and Stages Questionnaire, 3rd edition*; BSID-III: *Bayley Scales of Infants and Toddler Development – 3rd edition*; BISQ: *Brief Infant Sleep Questionnaire;* BPSC: *Baby Pediatric Symptom Checklist*; *CFCS = communication function classification scale*; DDST: Denver Developmental *Screening Test II;* EABR: Electrical Auditory Brainstem Response; EADCS *= eating and drinking ability classification scale;* GMA: *General Movements Assessment;* GMFM-66: *Gross Motor Function Measure;* GMFCS: *Gross Motor Function Classification System;* HINE: *Hammersmith Infant Neurological Examination*; MACS*: manual ability classification scale*, MOS*: Motor Optimality Score;* MSEL: *Mullen Scales of Early Learning;* PEDI: *Pediatric Evaluation of Disability Inventory*; PPSC: *Preschool Pediatric Symptom Checklist;* SD: *standard deviation; SWYC:* Survey of Wellbeing of Young Children. WIDEA: *Warner Initial Developmental Evaluation of Adaptive and Functional Skills.* | | | | | |

**References**

van der Linden, V., Pessoa, A., Dobyns, W., Barkovich, A. J., Júnior, H. V., Filho, E. L., et al. (2016). Description of 13 Infants Born During October 2015-January 2016 With Congenital Zika Virus Infection Without Microcephaly at Birth - Brazil. *MMWR. Morbidity and mortality weekly report*, *65*(47), 1343–1348. doi: 10.15585/mmwr.mm6547e2

Satterfield-Nash, A., Kotzky, K., Allen, J., Bertolli, J., Moore, C. A., Pereira, I. O., et al. (2017). Health and Development at Age 19-24 Months of 19 Children Who Were Born with Microcephaly and Laboratory Evidence of Congenital Zika Virus Infection During the 2015 Zika Virus Outbreak - Brazil, 2017. *MMWR. Morbidity and mortality weekly report*, 66(49), 1347–1351. doi: 10.15585/mmwr.mm6649a2

França, T., Medeiros, W. R., Souza, N. L., Longo, E., Pereira, S. A., França, T., et al. (2018). Growth and Development of Children with Microcephaly Associated with Congenital Zika Virus Syndrome in Brazil. *International journal of environmental research and public health*, 15(9), 1990. doi: 10.3390/ijerph15091990

Alves, L. V., Paredes, C. E., Silva, G. C., Mello, J. G., & Alves, J. G. (2018). Neurodevelopment of 24 children born in Brazil with congenital Zika syndrome in 2015: a case series study. *BMJ open*, *8*(7), e021304. doi: 10.1136/bmjopen-2017-021304

Ferreira, H., Schiariti, V., Regalado, I., Sousa, K. G., Pereira, S. A., Fechine, C., et al. (2018). Functioning and Disability Profile of Children with Microcephaly Associated with Congenital Zika Virus Infection. *International journal of environmental research and public health*, 15(6), 1107. doi: 10.3390/ijerph15061107

Wheeler, A. C., Ventura, C. V., Ridenour, T., Toth, D., Nobrega, L. L., Silva de Souza Dantas, et al. (2018). Skills attained by infants with congenital Zika syndrome: Pilot data from Brazil. *PloS one*, 13(7), e0201495. doi: 10.1371/journal.pone.0201495

Marques, F., Teixeira, M., Barra, R. R., de Lima, F. M., Dias, B., Pupe, C., et al. (2019). Children Born With Congenital Zika Syndrome Display Atypical Gross Motor Development and a Higher Risk for Cerebral Palsy. *Journal of child neurology*, *34*(2), 81–85. doi: 10.1177/0883073818811234

Carvalho, A., Brites, C., Mochida, G., Ventura, P., Fernandes, A., Lage, M. L., et al. (2019). Clinical and neurodevelopmental features in children with cerebral palsy and probable congenital Zika. *Brain & development*, *41*(7), 587–594. doi: 10.1016/j.braindev.2019.03.005

Carvalho, A. L., Ventura, P., Taguchi, T., Brandi, I., Brites, C., & Lucena, R. (2020). Cerebral Palsy in Children With Congenital Zika Syndrome: A 2-Year Neurodevelopmental Follow-up. *Journal of child neurology*, 35(3), 202–207. doi: 10.1177/0883073819885724

Lima DLP, Correia MLGCD, Monteiro MG, Ferraz KM, Wiesiolek CC. (2019) Analysis of the functional performance of infants with congenital zika syndrome: a longitudinal study. *Fisioter Pesqui*; 26(2): 145–50. doi: 10.1590/1809-2950/18001626022019

Vianna, R., Lovero, K. L., Oliveira, S. A., Fernandes, A. R., Santos, T., Lima, L., et al. (2019). Children Born to Mothers with Rash During Zika Virus Epidemic in Brazil: First 18 Months of Life. *Journal of tropical pediatrics*, 65(6), 592–602. doi: 10.1093/tropej/fmz019

Einspieler, C., Utsch, F., Brasil, P., Panvequio Aizawa, C. Y., Peyton, C., Hydee Hasue, R., et al. (2019). Association of Infants Exposed to Prenatal Zika Virus Infection With Their Clinical, Neurologic, and Developmental Status Evaluated via the General Movement Assessment Tool. *JAMA network open*, *2*(1), e187235. doi: 10.1001/jamanetworkopen.2018.7235

Wheeler, A. C., Toth, D., Ridenour, T., Lima Nóbrega, L., Borba Firmino, R., Marques da Silva, C., et al. (2020). Developmental Outcomes Among Young Children With Congenital Zika Syndrome in Brazil. *JAMA network open*, *3*(5), e204096. doi: 10.1001/jamanetworkopen.2020.4096

Melo, A., Gama, G. L., Da Silva Júnior, R. A., De Assunção, P. L., Tavares, J. S., Da Silva, M. B., et al. (2020). Motor function in children with congenital Zika syndrome. *Developmental medicine and child neurology*, *62*(2), 221–226. doi: 10.1111/dmcn.14227

Ventura, P. A., C Lage, M. L., L de Carvalho, A., S Fernandes, A., B Taguchi, T., & Nascimento-Carvalho, C. M. (2020). Early Gross Motor Development Among Brazilian Children with Microcephaly Born Right After Zika Virus Infection Outbreak. *Journal of developmental and behavioral pediatrics: JDBP*, *41*(2), 134–140. doi: 10.1097/DBP.0000000000000722

Bertolli, J., Attell, J. E., Rose, C., Moore, C. A., Melo, F., Staples, J. E., etal. (2020). Functional Outcomes among a Cohort of Children in Northeastern Brazil Meeting Criteria for Follow-Up of Congenital Zika Virus Infection. *The American journal of tropical medicine and hygiene*, 102(5), 955–963. doi: 10.4269/ajtmh.19-0961

Pereira, H., Dos Santos, S. P., Amâncio, A., de Oliveira-Szejnfeld, P. S., Flor, E. O., de Sales Tavares, J., et al. (2020). Neurological outcomes of congenital Zika syndrome in toddlers and preschoolers: a case series. *The Lancet. Child & adolescent health*, *4*(5), 378–387. doi: 10.1016/S2352-4642(20)30041-9

Sobral da Silva, P. F., Eickmann, S. H., Arraes de Alencar Ximenes, R., Ramos Montarroyos, U., de Carvalho Lima, M., Turchi Martelli, C. M., et al. (2020). Pediatric neurodevelopment by prenatal Zika virus exposure: a cross-sectional study of the Microcephaly Epidemic Research Group Cohort. *BMC pediatrics*, *20*(1), 472. doi: 10.1186/s12887-020-02331-2

Takahasi, E., Alves, M., Ribeiro, M., Souza, V., Simões, V., Borges, M., et al. (2021). Gross Motor Function in Children with Congenital Zika Syndrome. *Neuropediatrics*, *52*(1), 34–43. doi: 10.1055/s-0040-1718919

Frota, L., Sampaio, R. F., Miranda, J. L., Brasil, R., Gontijo, A., Mambrini, J., et al. (2020). Children with congenital Zika syndrome: symptoms, comorbidities and gross motor development at 24 months of age. *Heliyon*, *6*(6), e04130. doi: 10.1016/j.heliyon.2020.e04130

García-Boyano, M., García-Segovia, R., Fernández-Menéndez, A., Pérez, Y., Bustamante-Amador, J., Layana-Coronel, M., et al. (2020). Long-Term Outcomes of Infants with Congenital Zika Virus Infection in Ecuador: A Retrospective Longitudinal Study. *Journal of tropical pediatrics*, fmaa066. Advance online publication. doi: 10.1093/tropej/fmaa066

Campos, T., Schiariti, V., Gladstone, M., Melo, A., Tavares, J. S., Magalhães, A. G., et al. (2020). How congenital Zika virus impacted my child's functioning and disability: a Brazilian qualitative study guided by the ICF. *BMJ open*, *10*(12), e038228. doi: 10.1136/bmjopen-2020-038228

Cavalcante, T. B., Ribeiro, M., Sousa, P., Costa, E., Alves, M., Simões, V., et al. (2021). Congenital Zika syndrome: Growth, clinical, and motor development outcomes up to 36 months of age and differences according to microcephaly at birth. *International journal of infectious diseases*, *105*, 399–408. doi: 10.1016/j.ijid.2021.02.072
