# Supplementary material for: Neurodevelopment in Children Exposed to Zika in utero: Clinical and Molecular Aspects
Source: Front Genet. 2022 Mar 8;13:758715. doi: 10.3389/fgene.2022.758715 (PMC8957982; doi:10.3389/fgene.2022.758715)
Supplement: Supplementary file 3 [file Table3.docx]

| **Supplemental Table 3. Development of children exposed to ZIKV *in-utero* without congenital zika syndrome** | | | | | |
| --- | --- | --- | --- | --- | --- |
| **Study Design / reference / Study location** | **Sample size**  **CZS or ZIKV Exposure vs No CZS or Controls** | **Zika lab confirmation** | **Age of evaluation*** | **Assessment** | **Main Outcomes** |
| Case Series.  No control group  Lopes Moreira et al., (2018)  Rio de Janeiro, Brazil | 131  (This study does not identify or separate children with CZS from those without) | Yes | 12-18m | Brain Imaging in 115  Eye and Hearing 112, BSID-III in 104 | Abnormal findings on neuroimaging 34%. Of these, 11% had structural abnormalities.  Children with normal brain imaging were 20% less likely to have a Bayley-III score 2 SD below the mean (±SD) score than those with abnormal brain imaging. |
| Cohort study.  Control group.  Einspieler et al., (2019)  Rio de Janeiro and Belo Horizonte, Brazil | 111 prenatally exposed to ZKV: 35 CZS, 76 without CZS  and 333 control group matched for sex, GA | No (Clinical criteria) | 9-20 wks  12 m | GMA  MOS  BSID-III | 333 control infants had normal general movements and neurotypical development.  76 Infants prenatally exposed without microcephaly: 84% had normal general movements and as group scored significantly lower on the MOS than neurotypical controls. GMA was a good predictor of development at 12 months. BSID-III at 12 months was abnormal in 10 children (18%) in 5 of them BSID 70-85, and in 4 <70. |
| Cohort study: ZIKV exposed in-utero  No control group  Nielsen-Saines  et al., (2019)  Rio de Janeiro, Brazil | 216 children of ZIKV infected mothers (only 6 children could have CZS) | Yes | 7-32 m | BSID-III  HINE  Complete eye and hearing exams | Prematurity 13%. Microcephaly in 6 (3.5 %).  31% below-average neurodevelopment and/or abnormal eye or hearing assessments.  146 children assessed with BSID-III: 71.3% normal development. A score between 84–70 in 28% and a score <70 in at least one domain in the 12%. Language function altered in 35%. Autism in 3 children  An earlier gestational age at the time of ZIKV infection was a significant predictor of below average neurodevelopment. |
| Cross-sectional  No control group  Cardoso et al., (2019)  Rio de Janeiro, Brazil. | 19 children of ZIKV infected mothers | NR | 1-7 m | Neuro exam  AIMS  DDST-II | Only one child had a normal neurological evaluation. All others (18) had some signs of developmental abnormality: Hypotonia (2), hypertonia (16), Ataxia (10), dyskinesia (8), irritability (8), postural asymmetry (6).  AIMS: normal 12 (63%); suspect (4); delay (3); DDST-II: normal 12 (63%), suspect 7 (37%). |
| Cross-sectional:  Valdes et al., (2019)  Puerto Rico | 65 infants of ZIKV infected mothers  36 (55.4%) ZIKV negative and 29 (44.6%) ZIKV positive | Yes | 8.98±3.19 m  (9-12) | MSEL | General cognitive and motor scores did not differ between ZIKV positive and ZIKV negative, except for receptive language score. ZIKV+ and Hurricane Maria exposure were associated with lower receptive language. |
| Cohort study.  No control group  Bertolli et al., (2020)  Pernambuco, Brazil | 43 infants with anthropometric and laboratory criteria vs 77 with only laboratory or anthropometric criteria | Yes | Median age 23  (19-26) | HINE  ASQ3 | Infants with anthropometric and laboratory criteria: developmental delay (79%). Severe motor impairment (61%), cerebral palsy (58%). Impaired response to auditory and visual stimuli: 54% and 49, respectively. Ocular abnormalities: retinal (16%); abnormal fixation and following (37%). Seizure screening positive (43%)  Infants with only laboratory criteria or anthropometric criteria: Developmental delay mild/moderate (46%); severe (5%). |
| Case-series  No control group  Faiçal et al., (2019)  Salvador, Brazil | 29 children of ZIKV infected mothers | Yes | 18.2 ±3.8 | BSID-III  Ophthalmological evaluation | Delay in at least one of the BSID-III scale domains (34%), language delay (31%). Language function was the most impaired domain among the children evaluated.  Cognitive: mean 102.2 ±16.9. Range 70-145. < 70: 0; 70-84: 13.8%. Language: mean 93.8±18.5. Range 47-135. < 70: 2, 70-84: 24%. Motor: mean 103.5±12.0. Range 79-127. < 70: 0, 70-84: 3.4%.  Ophthalmological evaluations: no abnormalities identified. |
| Cohort study  No control group  Mulkey et al., (2020)  Atlántico Department, Colombia, and Washington  DC, USA | 77 children of ZIKV infected mothers  70 No CZS  7 CZS | Yes | 4-18 m | WIDEA  AIMS | Of 70 without CZS, 40 (57%) infants were evaluated between 4 and 8 months of age and 60 (86%) assessed between 9 and 18 months of age.  Head circumference z scores no decline over time. The median WIDEA total score was 60.5 (58-62 IQR) at time point 1 and 102.5 (83.3-123.8) at time point 2 in the 70 infants. Multidomain neurodevelopmental assessment scores deviated from normal scores as the children became older. |
| Cross-sectional:  Control group  Gerzson et al., (2020)  Tangará da Serra, Brazil | 17 children of ZIKV infected mothers  Control group 20 | Yes | 18-29 | BSID-III | Cognitive, motor and language scores: similar to 20 non-exposed infants of the same setting and same age. Only one child in the ZIKVG presented low cognitive score, the same in the control group. |
| Case-series  No control group  Peçanha et al., (2020)  Rio de Janeiro, Brazil | 84 children of ZIKV infected mothers | Yes | 9.7±3.1 (6-18)  15.3±3.1 (12-24) | BSID-III | Assessment at 9.7m: language (12%) or motor (3.5%) delay. None children had two or more domains affected simultaneously.  Assessment at 15.3 m: delay in one of the three domains (50%): cognition (5%), language (37%), motor performance (24%). 7 children at least two domains affected. Abnormalities were mainly in the language domain during the first two years of life. |
| Cross-Sectional  Control group  Sobral da Silva et al. 2020.,  Recife, Pernambuco, Brazil | 274 children who were born in epidemic outbreak  134 CZS  94 no CZS  46 Control group | Yes | 10-45 | SWYC  BPSC  PPSC | The 94 prenatally exposed babies without CZS showed a risk frequency of developmental delay similar to the control group but 2.2% showed epilepsy. |
| Case-series  No control group  Abtibol-Bernardino et al., (2020)  Manaus, Amazonas, Brazil | 26 children of ZIKV infected mothers | Yes | 37.8 ± 3. 95m (25–42) | BSID-III | 34.6% presented abnormal results: mild delays 26.9%, moderate motor delays 3.8% and severe language delays 3.8%.  4 (15%) had one abnormal domain, four (15%) two abnormal domains and one (3.8%) three abnormal domains. The most impaired domain was language, with an alteration in 30.7% (8/26) of children. Alterations of motor skills were present in 19.2% (5/26). |
| Cross-sectional study  Power et al., 2020  Rio de Janeiro, Brazil | 163 children of ZIKV infected mothers  51 microcephaly (CZS)  112 normocephalic at birth. | Yes | 19.6m  (4.9 - 40.1) | BSID-III  performed only in all normocephalic children.  In microcephaly not routinely undertaken | 112 normocephalic infants: mean composite language score of 90.3±13.1 (range 47 - 115). Mean composite motor score of 95.3±12.4, (range 50 - 124) and mean composite cognitive score of 102.8±13.5 (range 65 – 145). 26% presented severe delay for the language domain, 20% severe delay for the motor domain and 11% severe delay for the cognitive domain.  Odds of microcephaly were higher in families with lower household income and higher household crowding. Maternal secondary and higher education appeared to have a protective effect for microcephaly compared to primary education. |
| Cohort study  Blackmon et al., 2020  Grenada | 71children of ZIKV-uninfected mothers  71 children of ZIKV infected mothers | Yes | 24 m | Pediatric epilepsy screening questionnaire  Videoelectroencepha-lography | Epilepsy in 2/71 children of ZIKV infected mothers (IR: 2.8%; 95% CI: 0.34–9.81%) and 0/71 in children of ZIKV-uninfected mothers (IR: 0.00; 95% CI: 0.00–5.36%). Global epilepsy incidence rates range from 0.10% to 0.15% in the first year of life. |
| Case series  No control group  Andrade et al., 2021  Rio de Janeiro. Brazil | 96 children of ZIKV infected mothers | Yes | 12-18 m | BSID-III | 35.4% scored below the normal range in at least one Bayley-III domain. The majority (91.2%) of the infants with delayed scores presented language delay, which was not associated with the gestational age at exposure. Receptive language was more affected than expressive language (27.0% vs 19.8%). Significant, association between the head circumference Z-score at birth and language delay. |
| Case series  No control group  Aizawa et al., 2020  Sao Paulo, Brazil | 31 children of ZIKV infected mothers | Yes | 33–38 m 36±1,24 m | BSID-III | Most children (74%) had average neurodevelopmental scores and 8 children (26%) presented delay in some domain. Language was the most affected: 7 (22.6%) had a delay in this domain (2 severe delay). Moderate delay was detected in the cognitive (3.2%) and motor (10%) domains. Maternal illness in the third trimester of pregnancy and later gestational age at birth were associated with higher Bayley-III scores |
| Case-series  No control group  Cardona-Ospina et al., 2021  Risaralda, Colombia | 16 normocephalic | Yes | 28 m  (23-31) | Abbreviated neurodevelopment scale 2 validated for Colombia | Five patients presented post-natal microcephaly and six patients developed macrocephaly. Patients with a normal head circumference had normal neurodevelopment. 15/16 had normal neurodevelopment, only one patient with microcephaly persisted with impairment of the neurodevelopment at the end of follow-up. |
| Cohort study  Grant et al., 2021  Guadeloupe, Martinique, and French Guiana | 156 of ZIKV infected mothers vs  79 without ZIKV exposure | Yes | 24 m (±1 m) | ASQ3  M-CHAT  French MacArthur Inventory Scales (IFDC) for French language acquisition. | 15.4% ZIKV-exposed toddlers and 25.3% ZIKV-unexposed toddlers had an ASQ result below the reference − 2SD cut-off (P = 0.10) for at least one of the five ASQ dimensions.  *In utero* ZIKV exposure and sex were identified as independent predictors of an ASQ score for communication below the − 2SD cut-off.  No differences in M-CHAT and in mean language acquisition. |
| Cohort study  Familiar et al., 2021  Yucatan, Mexico | 60 of ZIKV infected mothers vs  60 without ZIKV exposure | Yes | Approximately 6 m | MSEL  FTII using automated eye-tracking instrumentation | All MSEL subscale scores, except expressive language, significantly lower among ZIKV exposed children compared to controls, including the overall standard composite (80 ± 10 vs. 87 ± 7.4, respectively).  No statistical differences in FTII eye-tracking measures of fixation and gaze. |
| Age of evaluation: mean ± standard deviation or median (range). m=months, wks= weeks, y= years. AIMS - *Alberta Infant Motor Scale;* ICF: *The International Classification of Functioning Disability and Health*; ASQ-3 *Ages and Stages Questionnaire, 3rd edition*; BSID-III: *Bayley Scales of Infants and Toddler Development – 3rd edition*; BISQ: *Brief Infant Sleep Questionnaire;* BPSC: *Baby Pediatric Symptom Checklist*; *CFCS = communication function classification scale*; DDST: Denver Developmental *Screening Test II;* EABR: Electrical Auditory Brainstem Response; EADCS *= eating and drinking ability classification scale;* FTII = Fagan test of infant intelligence; GMA: *General Movements Assessment;* GMFM-66: *Gross Motor Function Measure;* GMFCS: *Gross Motor Function Classification System;* HINE: *Hammersmith Infant Neurological Examination*; IQR: interquartile range, MACS*: manual ability classification scale*, M-CHAT=Modified Checklist for Autism on Toddlers; MOS*: Motor Optimality Score;* MSEL: *Mullen Scales of Early Learning;* NR: *Non reported*; PEDI: *Pediatric Evaluation of Disability Inventory*; PPSC: *Preschool Pediatric Symptom Checklist;* SD: *standard deviation; SWYC:* Survey of Wellbeing of Young Children. WIDEA: *Warner Initial Developmental Evaluation of Adaptive and Functional Skills.* | | | | | |

**References**

Lopes Moreira, M. E., Nielsen-Saines, K., Brasil, P., Kerin, T., Damasceno, L., Pone, M., et al. (2018). Neurodevelopment in Infants Exposed to Zika Virus In Utero. *The New England journal of medicine*, *379*(24), 2377–2379. doi: 10.1056/NEJMc1800098

Einspieler, C., Utsch, F., Brasil, P., Panvequio Aizawa, C. Y., Peyton, C., Hydee Hasue, R., et al. (2019). Association of Infants Exposed to Prenatal Zika Virus Infection With Their Clinical, Neurologic, and Developmental Status Evaluated via the General Movement Assessment Tool. *JAMA network open*, *2*(1), e187235. doi: 10.1001/jamanetworkopen.2018.7235

Nielsen-Saines, K., Brasil, P., Kerin, T., Vasconcelos, Z., Gabaglia, C. R., Damasceno, L., et al. (2019). Delayed childhood neurodevelopment and neurosensory alterations in the second year of life in a prospective cohort of ZIKV-exposed children. *Nature medicine*, *25*(8), 1213–1217. doi: 10.1038/s41591-019-0496-1

Cardoso, T. F., Jr, Santos, R., Corrêa, R. M., Campos, J. V., Silva, R. B., Tobias, C. C., et al. (2019). Congenital Zika infection: neurology can occur without microcephaly. *Archives of disease in childhood*, *104*(2), 199–200. doi: 10.1136/archdischild-2018-314782

Valdes, V., Zorrilla, C. D., Gabard-Durnam, L., Muler-Mendez, N., Rahman, Z. I., Rivera, D., et al. (2019). Cognitive Development of Infants Exposed to the Zika Virus in Puerto Rico. *JAMA network open*, *2*(10), e1914061. doi: 10.1001/jamanetworkopen.2019.14061

Bertolli, J., Attell, J. E., Rose, C., Moore, C. A., Melo, F., Staples, J. E., etal. (2020). Functional Outcomes among a Cohort of Children in Northeastern Brazil Meeting Criteria for Follow-Up of Congenital Zika Virus Infection. *The American journal of tropical medicine and hygiene*, 102(5), 955–963. doi: 10.4269/ajtmh.19-0961

Faiçal, A. V., de Oliveira, J. C., Oliveira, J., de Almeida, B. L., Agra, I. A., Alcantara, L., et al. (2019). Neurodevelopmental delay in normocephalic children with in utero exposure to Zika virus. *BMJ paediatrics open*, *3*(1), e000486. doi: 10.1136/bmjpo-2019-000486

Mulkey, S. B., Arroyave-Wessel, M., Peyton, C., Bulas, D. I., Fourzali, Y., Jiang, J., et al. (2020). Neurodevelopmental Abnormalities in Children With In Utero Zika Virus Exposure Without Congenital Zika Syndrome. *JAMA pediatrics*, 174(3), 269–276. doi: 10.1001/jamapediatrics.2019.5204

Gerzson, L. R., de Almeida, C. S., Silva, J., Feitosa, M., de Oliveira, L. N., & Schuler-Faccini, L. (2020). Neurodevelopment of Nonmicrocephalic Children, After 18 Months of Life, Exposed Prenatally to Zika Virus. *Journal of child neurology*, *35*(4), 278–282. doi: 10.1177/0883073819892128

Peçanha, P. M., Gomes Junior, S. C., Pone, S. M., Pone, M., Vasconcelos, Z., Zin, A., et al. (2020). Neurodevelopment of children exposed intra-uterus by Zika virus: A case series. *PloS one*, *15*(2), e0229434. doi: 10.1371/journal.pone.0229434

Sobral da Silva, P. F., Eickmann, S. H., Arraes de Alencar Ximenes, R., Ramos Montarroyos, U., de Carvalho Lima, M., Turchi Martelli, C. M., et al. (2020). Pediatric neurodevelopment by prenatal Zika virus exposure: a cross-sectional study of the Microcephaly Epidemic Research Group Cohort. *BMC pediatrics*, *20*(1), 472. doi: 10.1186/s12887-020-02331-2

Abtibol-Bernardino, M. R., de Almeida Peixoto, L., de Oliveira, G. A., de Almeida, T. F., Rodrigues, G., Otani, R. H., et al. (2020). Neurological Findings in Children without Congenital Microcephaly Exposed to Zika Virus in Utero: A Case Series Study. *Viruses*, *12*(11), 1335. doi: 10.3390/v12111335

Power, G. M., Francis, S. C., Sanchez Clemente, N., Vasconcelos, Z., Brasil, P., et al. (2020). Examining the Association of Socioeconomic Position with Microcephaly and Delayed Childhood Neurodevelopment among Children with Prenatal Zika Virus Exposure. *Viruses*, 12(11), 1342. doi: 10.3390/v12111342

Blackmon, K., Waechter, R., Landon, B., Noël, T., Macpherson, C., Donald, T., et al. (2020). Epilepsy surveillance in normocephalic children with and without prenatal Zika virus exposure. *PLoS neglected tropical diseases*, *14*(11), e0008874. doi: 10.1371/journal.pntd.0008874

Andrade, L. M., Baker Meio, M. D., Gomes, S. C., Jr, Souza, J. P., Figueiredo, M. R., Costa, et al. (2021). Language delay was associated with a smaller head circumference at birth in asymptomatic infants prenatally exposed to the Zika virus. *Acta paediatrica*, 10.1111/apa.15878. Advance online publication. doi: 10.1111/apa.15878

Aizawa, C., Caron, D., Souza, C. B., Kozima, P., Damasceno, L., Einspieler, C., et al. (2021). Neurodevelopment in the third year of life in children with antenatal ZIKV-exposure. *Revista de saude publica*, 55, 15. doi: 10.11606/s1518-8787.2021055002798

Cardona-Ospina, J. A., Zapata, M. F., Grajales, M., Arias, M. A., Grajales, J., Bedoya-Rendón, H. D., et al. (2021). Physical Growth and Neurodevelopment of a Cohort of Children after 3.5 Years of Follow-up from Mothers with Zika Infection during Pregnancy-Third Report of the ZIKERNCOL Study. *Journal of tropical pediatrics*, *67*(2), fmab032. doi: 10.1093/tropej/fmab032

Grant, R., Fléchelles, O., Tressières, B., Dialo, M., Elenga, N., Mediamolle, N., et al. (2021). In utero Zika virus exposure and neurodevelopment at 24 months in toddlers normocephalic at birth: a cohort study. *BMC medicine*, *19*(1), 12. doi: 10.1186/s12916-020-01888-0

Familiar, I., Boivin, M., Magen, J., Azcorra, J. A., Phippen, C., Barrett, E. A., et al. (2021). Neurodevelopment outcomes in infants born to women with Zika virus infection during pregnancy in Mexico. *Child: care, health and development*, *47*(3), 311–318. doi: 10.1111/cch.12842
