# Supplementary material for: Neurodevelopment in Children Exposed to Zika in utero: Clinical and Molecular Aspects
Source: Front Genet. 2022 Mar 8;13:758715. doi: 10.3389/fgene.2022.758715 (PMC8957982; doi:10.3389/fgene.2022.758715)
Supplement: Supplementary file 4 [file Table4.docx]

| **Supplementary Table 4.** Summary of studies using biosystems approach and its main findings | | | | | | | | |
| --- | --- | --- | --- | --- | --- | --- | --- | --- |
| **Author (year)** | | **Aim** | **Cell type** | **Molecular analyses** | **System biology tools** | **Molecular results** | **Application of system biology analyses** | **Relationship of molecular results and neurological outcomes** |
| Aguiar *et al.* (2020)^1^ | | To perform a multiomics analysis in postmortem brains of neonates with CZS | Postmortem brains | Exome  Transcriptome  Proteome | NetworkAnalyst, STRING, and Cytoscape | Reduced expression (RNA and protein) of collagen genes and upregulation of cell adhesion genes | To integrate the exome, transcriptome and proteome findings | Collagen genes have been previously associated to arthrogryposis and cell adhesion factors to neuronal migration and axon guidance |
| Anderson *et al.* (2020)^2^ | | To determine whether ZIKV modulates host cell epigenetic profiles *in vivo* | Blood cells | Genome-wide DNA methylation profiling | Packages in R | Altered host methylation patterns, especially at *RABGAP1L* - important in brain development -, at viral host immunity genes *MX1* and *ISG15*, and in an epigenetic module containing the causal microcephaly gene *MCPH1* | To identify differentially regulated modules of genes that may be contributing to the microcephaly phenotype | Given the importance of the affected genes to brain development and antiviral response, their altered methylation could the clinical signs of CZS |
| Park *et al*. (2020)^4^ | | To describe molecular alterations due the ZIKV infection *in vitro* | hPNs, hNCCs, mDCs | Transcriptome | PPI in STRING, Cytoscape | The deregulated genes were related to DNA repair and prolactin signaling as well as the interferon signaling, neuroinflammation, and cell cycle pathways | To perform a protein-protein interaction analysis of the DGE genes identified in RNA-seq | Neuronal cell damage occurred through up-regulation of neuroinflammation and down-regulation of the DNA repair system |
| Saade *et al.* (2020)^5^ | | To investigate the interaction between ZikV-NS5 protein with the interactome of hNPCs | hNPCs | Zika-host proteome interaction | PPI network, Gene Ontology enrichment | Enrichment of cytoskeleton, especially microtubule, host proteins that interact with ZikV-NS5 | To identify the Gene Ontologies of the human proteins that interact with ZikV-NS5, according to the human fetal brain library | The identification of these proteins might help to understand how ZIKV impacts neurogenesis |
| Souza *et al*. (2020)^6^ | | *In silico* protocol: key genes and pathways for emerging virus disease pathogenesis | Human neural cells infected with ZIKV | Transcriptome | PPI network: Gephi, STRING, Cytoscape | 30 DEGs (24 up and 6 down) associated to endoplasmic reticulum stress and DNA replication. Neurogenesis and/or apoptosis | Integrative analysis of the up and down regulated genes | Downregulation of *CENPF*, *MCM2, MCM4, MCM6* genes (DNA replication on neocortex) possibly related to microcephaly |
| Tabari *et al.* (2020)^7^ | | To profile the host transcriptome after ZIKV infection | hNSC infected with ZIKV Uganda and French Polinesia strains | Transcriptome and miRnome | Reactome signaling pathways and networks | Upregulation of genes included in antiviral infection signaling, ER stress, and unfolded proteins pathways. Downregulation of signal transduction, cell cycle, and gene expression related pathways. | To identify the signaling pathways the perturbed genes are associated with | *ASNS*, a gene previously associated to microcephaly, was perturbed by both strains |
| Beys-da-Silva *et al*. (2019)^8^ | | To describe proteome alterations due the ZIKV infection *in vitro* | hMSC | Shotgun proteomics | PPI in STRING, Cytoscape | Reprogramming of the metabolic machinery and potential inhibition of autophagy, neurogenesis, and immune response; Proteins related to Alzheimer’s disease, autism spectrum disorder, amyotrophic lateral sclerosis, and Parkinson’s disease, had their expression altered due to ZIKV | To associate deregulated genes with human brain diseases | The molecular pathways affected by ZIKV in hMSC are related to brain diseases |
| Dang *et al*. (2019)^9^ | | Role of miRNAs in ZIKV pathogenesis and microcephaly | hNSCs | mRNA and miRNA transcriptomes | miRNA-mRNA interactions Cytoscape, | Upregulation of some miRNA (e.g. let-7c and miR-124–3p), repressors cell cycle, stem cell maintenance, and neurogenesis genes | To analyze miRNA-mRNA interactions in hNSC infected cells | ZIKV upregulated miR124–3p and downregulated the transferrin receptor (*TFRC*) in ZIKV-infected hNSCs - NSC maintenance dysregulated |
| Lima *et al*. (2019)^10^ | | Transcriptional changes induced by ZIKV infection *in vitro* | hiNPCs | Transcriptome | PPI in  STRING, Cytoscape | Induction of type-I interferons (IFN-I) IFN-Is stimulated genes (ISGs): cytokines and pro-apoptotic chemokines CXCL9 and CXCL10 | To identify specific networks induced by ZIKV in infected hiNPCs | Unbalanced and chronic local inflammatory response elicited by ZIKV infection, which contributes to damage to the fetal brain |
| Brahma *et al*. (2018)^11^ | To have a systems-level understanding of biological process affected by ZIKV in fetal brain | | Human fetal NSCs | Transcriptome | PPI in  STRING, Cytoscape | 613 downregulated genes: defense response to virus, receptor binding, laminin binding, extracellular matrix and endoplasmic reticulum. 471 upregulated: translation initiation, RNA binding | To identify candidate genes associated to ZIKV infection through a network-based analysis, assembling PPI networks | Pathways such as systemic lupus erythematosus, defense responses to virus, ribosome structure, chromosome, and platelets could be all involved in ZIKV microcephaly |
| Caires-Júnior *et al*. (2018)^12^ | Gene expression of cells from CZS-affected and CZS- unaffected twins | | hNPCs derived | Transcriptome | Networks of enriched GO in Metascape | 64 DEGs associated with regionalization, embryonic morphogenesis, embryo development and central nervous system development ontologies | To evaluate interactions between enriched gene ontologies | Differential expression of neural development genes (e.g. *DDIT4L*) may contribute to the different susceptibilities to the ZIKV^BR^ infection |
| Janssens *et al*. (2018)^13^ | ZIKV  infection in the neural DNA methylation | | Human NPCs, astrocytes, differentiated neurons | Methylome | *psygenetR*  package (R language) | ZIKV alters DNA methylation at specific gene loci implicated in the pathogenesis of brain disorders, especially neuropsychiatric disorders | Network associating genes differentially methylated and neuropsychiatric disorders | ZIKV-induced methylation changes suggest that infection during fetal development could lead to a spectrum of late-onset neuropsychiatric disorders |
| Garcez *et al*. (2017)^14^ | To describe molecular alterations due the ZIKV infection in vitro | | Human neurospheres derived from iPS | Transcriptome and proteome | PPI in STRING | Upregulation of response to viral replication, DNA damage, cell cycle arrest and apoptosis pathways, downregulation of neuronal differentiation | To create a interactome map of proteins and genes altered by ZIKV infection | ZIKV downregulates the neurogenesis and increases cell death in progenitor cells, which could be related to the ZIKV congenital syndrome |
| Moni *et al*. (2017)^15^ | | To understand the mechanism of Zika-associated disorders through infectome and diseasome analyses of ZIKV infection | hiPSCs and hNPCs | Transcriptome | Cytoscape | 341 genes upregulated: Protein processing in endoplasmic reticulum and transfer RNA biosynthesis signaling pathways; 588 genes downregulated: Cell cycle, DNA replication, and Fanconi anemia pathways | To analyze a network containing data related to ZIKV infectome, diseasome and associated comorbidities | Interlinked genes between ZIKV infection and other diseases indicates that metabolic, neurological, and cancer disease categories are possibly implicated in ZIKV infection and malformations |
| Rolfe *et al*. (2016)^16^ | | To analyzed the transcriptional changes induced by ZIKV infection *in vitro* | hNPCs | Transcriptome | Cytoscape, ClueGO app | Upregulated genes related to nucleic acid metabolism regulation pathway. Enrichment of numerous pro-inflammatory pathways. Downregulated genes related to chromosome segregation. | To provide a network of the biological processes associated to ZIKV infection | Enrichment of pro-inflammatory pathways causes CNS cytotoxic pro-inflammatory environment that could induce cell death. DGE association with neurological clinical phenotypes, such as microcephaly, epilepsy |
| Zhang *et al*. (2016)^17^ | | To describe molecular alterations due the ZIKV infection in vitro | hNPCs | Transcriptome | Gene-gene interaction networks GeneMania | The Asian ZIKV induced dysregulation of DNA replication and repair genes (e.g. *TP53*), and the upregulation of viral response genes, Type II interferon signaling, Toll-like receptor signaling and TNF signaling pathways | To evaluate gene-gene interactions that could be considered molecular signatures for the infection of different ZIKV strains | The p53 upregulation could play a pivotal role in the apoptosis in hNPCs |
| PPI: Protein-protein interaction; String: String database; Cytoscape: Cytoscape software; Gephi: Gephi software; ClueGO: ClueGO app; GO: Gene ontology; hiNPCs: Human induced neuroprogenitor cells; CZS: Congenital Zika Syndrome; NPCs: Neuroprogenitor cells; hNPCs: Human neuroprogenitor cells; iPS: induced pluripotent stem; hPNs: Peripheral neurons, hNCCs: neural crest cells; mDCs: myeloid dendritic cells; hMSC: Human mesenchymal stem cells; DGE: differential gene expressed. | | | | | | | | |

**References:**

1. Aguiar, R. S., Pohl, F., Morais, G. L., Nogueira, F., Carvalho, J. B., Guida, L., Arge, L., Melo, A., Moreira, M., Cunha, D. P., Gomes, L., Portari, E. A., Velasquez, E., Melani, R. D., Pezzuto, P., de Castro, F. L., Geddes, V., Gerber, A. L., Azevedo, G. S., Schamber-Reis, B. L., … Nakaya, H. I. (2020). Molecular alterations in the extracellular matrix in the brains of newborns with congenital Zika syndrome. *Science signaling*, **13(635),** eaay6736.
2. Anderson D, Neri JICF, Souza CRM, Valverde JG, De Araújo JMG, Nascimento MDSB, Branco RCC, Arrais NMR, Lassmann T, Blackwell JM, Jeronimo SMB. Zika Virus Changes Methylation of Genes Involved in Immune Response and Neural Development in Brazilian Babies Born With Congenital Microcephaly. *J Infect Dis* 2021; **223**(3): 435-440.
3. Park T, Kang MG, Baek SH, Lee CH, Park D. Zika virus infection differentially affects genome-wide transcription in neuronal cells and myeloid dendritic cells. *PLoS One* 2020; **15**(4): e0231049.
4. Saade, M., Ferrero, D. S., Blanco-Ameijeiras, J., Gonzalez-Gobartt, E., Flores-Mendez, M., Ruiz-Arroyo, V. M., Martínez-Sáez, E., Ramón Y Cajal, S., Akizu, N., Verdaguer, N., & Martí, E. (2020). Multimerization of Zika Virus-NS5 Causes Ciliopathy and Forces Premature Neurogenesis. *Cell stem cell*, **27(6),** 920–936.e8.
5. Souza GAP, Salvador EA, de Oliveira FR, Cotta Malaquias LC, Abrahão JS, Leomil Coelho LF. An in silico integrative protocol for identifying key genes and pathways useful to understand emerging virus disease pathogenesis. *Virus Res* 2020; **284**: 197986.
6. Tabari, D., Scholl, C., Steffens, M., Weickhardt, S., Elgner, F., Bender, D., Herrlein, M. L., Sabino, C., Semkova, V., Peitz, M., Till, A., Brüstle, O., Hildt, E., & Stingl, J. (2020). Impact of Zika Virus Infection on Human Neural Stem Cell MicroRNA Signatures. *Viruses*, **12(11),** 1219.
7. Beys-da-Silva WO, Rosa RL, Santi L, Berger M, Park SK, Campos AR, *et al*. Zika virus infection of human mesenchymal stem cells promotes differential expression of proteins linked to several neurological diseases. *Mol Neurobiol* 2019; **56**: 4708–17.
8. Dang JW, Tiwari SK, Qin Y, Rana TM. Genome-wide Integrative Analysis of Zika-Virus-Infected Neuronal Stem Cells Reveals Roles for MicroRNAs in Cell Cycle and Stemness. *Cell Rep* 2019; **27**(12): 3618–28.e5.
9. Lima MC, de Mendonça LR, Rezende AM, Carrera RM, Aníbal-Silva CE, Demers M, *et al*. The Transcriptional and Protein Profile From Human Infected Neuroprogenitor Cells Is Strongly Correlated to Zika Virus Microcephaly Cytokines Phenotype Evidencing a Persistent Inflammation in the CNS. *Front Immunol* 2019; **10**: 1928.
10. Brahma R, Gurumayum S, Naorem LD, Muthaiyan M, Gopal J, Venkatesan A. Identification of Hub Genes and Pathways in Zika Virus Infection Using RNA-Seq Data: A Network-Based Computational Approach. *Viral Immunol* 2018; **31**(4): 321–32.
11. Caires-Júnior LC, Goulart E, Melo US, Araujo BHS, Alvizi L, Soares-Schanoski A, *et al*. Discordant congenital Zika syndrome twins show differential in vitro viral susceptibility of neural progenitor cells [published correction appears in *Nat Commun* 2018 Mar 13; **9**(1):1114]. *Nat Commun* 2018; **9**(1):475.
12. Janssens S, Schotsaert M, Karnik R, Balasubramaniam V, Dejosez M, Meissner A, *et al*. Zika Virus Alters DNA Methylation of Neural Genes in an Organoid Model of the Developing Human Brain. *mSystems* 2018; **3**(1):e00219-17.
13. Garcez PP, Nascimento JM, de Vasconcelos JM, Madeiro da Costa R, Delvecchio R, Trindade P, *et al*. Zika virus disrupts molecular fingerprinting of human neurospheres. *Sci Rep* 2017; **7**: 40780.
14. Moni MA, Lio' P. Genetic Profiling and Comorbidities of Zika Infection. *J Infect Dis* 2017; **216**(6): 703–12.
15. Rolfe AJ, Bosco DB, Wang J, Nowakowski RS, Fan J, Ren Y. Bioinformatic analysis reveals the expression of unique transcriptomic signatures in Zika virus infected human neural stem cells. *Cell Biosci* 2016; **6**: 42.
16. Zhang F, Hammack C, Ogden SC, Cheng Y, Lee EM, Wen Z, *et al*. Molecular signatures associated with ZIKV exposure in human cortical neural progenitors. *Nucleic Acids Res* 2016; **44**(18): 8610–20.
